# Imaginary Poynting momentum: polarization topology and versatile optical manipulation

**DOI:** 10.1093/nsr/nwag171

**Published:** 2026-03-17

**Authors:** Yuchen Zhu, Yuzhi Shi, Tao He, Qinghua Song, Weijin Chen, Chengxing Lai, Zeyong Wei, Xiong Dun, Zhanshan Wang, Che Ting Chan, Cheng-Wei Qiu, Xinbin Cheng

**Affiliations:** Institute of Precision Optical Engineering, School of Physics Science and Engineering, Tongji University, Shanghai 200092, China; Technology Innovation Center of Mass Spectrometry for State Market Regulation, Center for Advanced Measurement Science, National Institute of Metrology, Beijing 100029, China; MOE Key Laboratory of Advanced Micro-Structured Materials, Shanghai 200092, China; Shanghai Institute of Intelligent Science and Technology, Tongji University, Shanghai 200092, China; Institute of Precision Optical Engineering, School of Physics Science and Engineering, Tongji University, Shanghai 200092, China; Technology Innovation Center of Mass Spectrometry for State Market Regulation, Center for Advanced Measurement Science, National Institute of Metrology, Beijing 100029, China; MOE Key Laboratory of Advanced Micro-Structured Materials, Shanghai 200092, China; Shanghai Institute of Intelligent Science and Technology, Tongji University, Shanghai 200092, China; Institute of Precision Optical Engineering, School of Physics Science and Engineering, Tongji University, Shanghai 200092, China; Technology Innovation Center of Mass Spectrometry for State Market Regulation, Center for Advanced Measurement Science, National Institute of Metrology, Beijing 100029, China; MOE Key Laboratory of Advanced Micro-Structured Materials, Shanghai 200092, China; Shanghai Institute of Intelligent Science and Technology, Tongji University, Shanghai 200092, China; Tsinghua Shenzhen International Graduate School, Tsinghua University, Shenzhen 518055, China; Institute of Precision Optical Engineering, School of Physics Science and Engineering, Tongji University, Shanghai 200092, China; Technology Innovation Center of Mass Spectrometry for State Market Regulation, Center for Advanced Measurement Science, National Institute of Metrology, Beijing 100029, China; MOE Key Laboratory of Advanced Micro-Structured Materials, Shanghai 200092, China; Shanghai Institute of Intelligent Science and Technology, Tongji University, Shanghai 200092, China; Institute of Precision Optical Engineering, School of Physics Science and Engineering, Tongji University, Shanghai 200092, China; Technology Innovation Center of Mass Spectrometry for State Market Regulation, Center for Advanced Measurement Science, National Institute of Metrology, Beijing 100029, China; MOE Key Laboratory of Advanced Micro-Structured Materials, Shanghai 200092, China; Shanghai Institute of Intelligent Science and Technology, Tongji University, Shanghai 200092, China; Institute of Precision Optical Engineering, School of Physics Science and Engineering, Tongji University, Shanghai 200092, China; Technology Innovation Center of Mass Spectrometry for State Market Regulation, Center for Advanced Measurement Science, National Institute of Metrology, Beijing 100029, China; MOE Key Laboratory of Advanced Micro-Structured Materials, Shanghai 200092, China; Shanghai Institute of Intelligent Science and Technology, Tongji University, Shanghai 200092, China; Institute of Precision Optical Engineering, School of Physics Science and Engineering, Tongji University, Shanghai 200092, China; Technology Innovation Center of Mass Spectrometry for State Market Regulation, Center for Advanced Measurement Science, National Institute of Metrology, Beijing 100029, China; MOE Key Laboratory of Advanced Micro-Structured Materials, Shanghai 200092, China; Shanghai Institute of Intelligent Science and Technology, Tongji University, Shanghai 200092, China; Institute of Precision Optical Engineering, School of Physics Science and Engineering, Tongji University, Shanghai 200092, China; Technology Innovation Center of Mass Spectrometry for State Market Regulation, Center for Advanced Measurement Science, National Institute of Metrology, Beijing 100029, China; MOE Key Laboratory of Advanced Micro-Structured Materials, Shanghai 200092, China; Shanghai Institute of Intelligent Science and Technology, Tongji University, Shanghai 200092, China; Department of Physics, The Hong Kong University of Science and Technology, Hong Kong 999077, China; Department of Electrical and Computer Engineering, National University of Singapore, Singapore 117583, Singapore; Institute of Precision Optical Engineering, School of Physics Science and Engineering, Tongji University, Shanghai 200092, China; Technology Innovation Center of Mass Spectrometry for State Market Regulation, Center for Advanced Measurement Science, National Institute of Metrology, Beijing 100029, China; MOE Key Laboratory of Advanced Micro-Structured Materials, Shanghai 200092, China; Shanghai Institute of Intelligent Science and Technology, Tongji University, Shanghai 200092, China

**Keywords:** imaginary Poynting momentum, polarization topological charge, optical force, advanced particle manipulation

## Abstract

The imaginary Poynting momentum (IPM), as an intrinsic yet mysterious property of light, is nearly as ubiquitous as its real counterpart. However, prior research has only traced its origin to intensity asymmetry and showcased its capability in particle manipulation using evanescent waves and structured light. Fundamentally, the interplay between the IPM and polarization topology remains unexplored, significantly restricting its potential applications. Here, we observe the high-order polarization topological charges (PTCs) in the IPM and expand their capabilities in versatile particle manipulation. PTCs originate from spatial distributions of linear polarizations in vector beams. When the PTC equals 1, the IPM can be utilized to rotate particles. Distinctively, for higher-order PTCs, rotational potential-well arrays with controllable rotation directions emerge to trap and rotate various numbers of particles. This work uncovers a holistic and complete understanding of the IPM by investigating its link to the polarization distribution. It also suggests a credible way to harness polarization-topology optical forces, offering significant potential for biophysical and quantum applications.

## Teaser

The team unveils the link between the imaginary Poynting momentum and the polarization topology charge by particle dynamics, offering a novel paradigm for particle manipulation.

## INTRODUCTION

**Figure 1.**
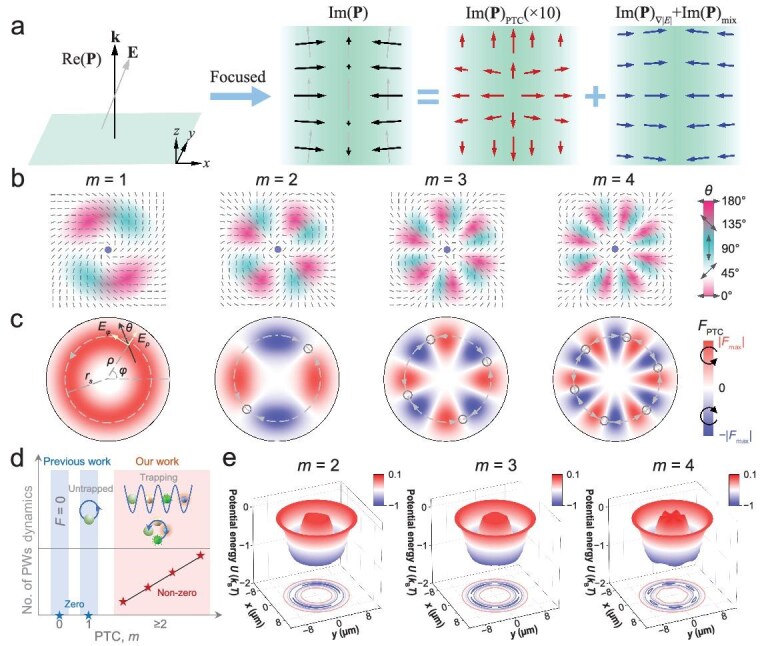
Physics of the IPM and its application in particle manipulation. (a) A plane wave after focusing can generate the IPM with three distinct components: |*E*|-asymmetry part, PTC-related part, and mixed term. (b) Polarization distributions along the circle with the maximum intensity, showing various PTCs. (c) PTOFs at different PTCs. The electric field in the ring can be decomposed into axial (*E_ρ_*) and azimuthal (*E_φ_*) components. Particles rotate at *m* = 1, while they can be trapped under the balance of positive and negative PTOFs at *m* > 1. The polarization angle *θ* is defined as the angle between the local polarization direction and the radial vector. (d) Milestones in experimental polarization-topology optical manipulation. Previous studies have shown that no optical force arises when the PTC *m* = 0, while the particle rotates when *m* = 1. Our work exploits the high-order polarization topology in the IPM, enabling multi-trapping and controlled rotation of the particle array. The number of potential wells also increases linearly with the PTC when *m* ≥ 2. (e) Simulation of potential wells at *m* = 2, 3, and 4. The number of potential wells increases linearly with the PTC, following the relationship 2(*m* − 1). The laser intensity in the circle is set to 1 mW/μm^2^.

Optical topology explores topological properties of light fields, finding numerous applications in lasers, sensing, particle manipulation, and quantum computing [1–8]. Topological charges describe singularities of various light properties, such as phase [9–12], polarization [13–17], intensity [18,19], etc. Among intrinsic and ubiquitous light properties, the imaginary Poynting momentum (IPM), as a counterpart of the real electromagnetic power, has been widely explored in evanescent waves [20–26], structured light [27–29], etc. Notably, the IPM is also present in optical fields with complex polarization states. For instance, in vector Lissajous beams, regions of positive and negative IPM values are spatially distributed with symmetry [30,31]. The primary obstacle hindering its practical applications is the limited understanding of the underlying physics. In fact, though the IPM has raised significant attention, its physics and applications are still in the infant stage. Most reported works rely on the electric-field (|*E*|, or intensity) asymmetry and overlook the topological characteristic of the IPM [20,28,32,33] (Fig. 1a), which, as demonstrated in this work, serves as a powerful approach for probing the IPM and expanding its applications.

Here, we uncover the complete understanding of the IPM and its link to the high-order polarization topological charges (PTCs, *m*) via topological optical forces and particle dynamics. PTCs originate from spatial distributions of linear polarizations. Intriguingly, the particle dynamics depend closely on the PTC. Particles can rotate clockwise or counter clockwise when *m* = 1. This is, in principle, similar to the optical rotation in an azimuthally polarized beam [34–39]. However, previous works fail to elaborate on the physics of the IPM. More significantly, potential wells emerge to trap particles at high-order PTCs (*m* > 1). The quantity of potential wells increases linearly with the PTC, following the relationship 2(*m* − 1). Those potential wells can also rotate simultaneously when changing the polarization state *θ*_0_ by mechanically rotating the half-wave plate.

Meanwhile, for polarization-dependent optical forces, previous studies have leveraged the spatial distribution of elliptical polarizations to manipulate particles [40–45]. Trapping achiral particles using spatial distributions of linear polarizations was recently theoretically proposed in evanescent waves [32,33]. So far, there lacks a theoretical foundation and an experimental demonstration of the high-order polarization-topology optical force (PTOF) with varying PTCs.

In this work, we present a holistic and generic theoretical framework for the IPM, elucidating its connection to electric-field asymmetry and polarization topology, and demonstrating its potential for versatile particle manipulation. We show that first and higher orders of PTCs induce the rotation and multiple trapping, respectively (Fig. 1b−e). This work offers a visual approach to investigate the IPM and drive its practical applications. It also showcases the pivotal role of polarization topology in optical manipulation, including trapping, rotation, and orbital motion of potential-well arrays.

## RESULTS

**Figure 2.**
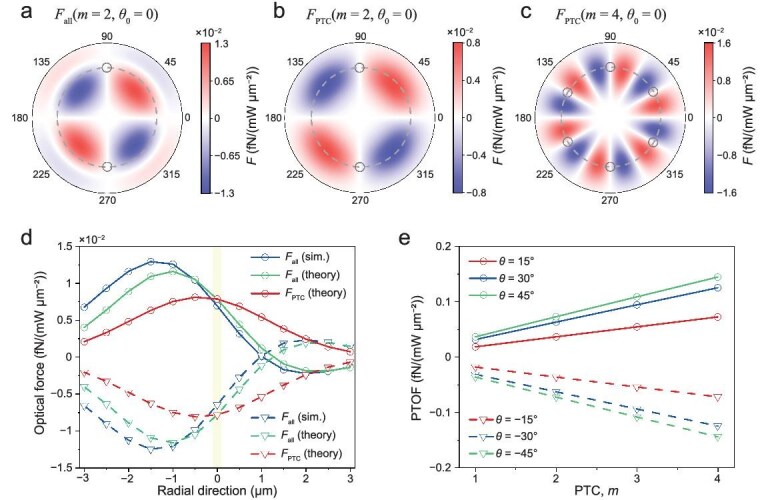
Simulation of optical forces from the IPM. (a) Simulated total optical force (*F*_all_) on the 100-nm nanoparticle using the Minkowski stress tensor in the doughnut-shaped beam [simulation radius (solid boundary), 9 μm; radius of the circle (dashed line), 6 μm] when *m* = 2. Opposite optical forces appear on both sides of the circle with the maximum intensity due to different gradients of electric fields. Simulated PTOFs (*F*_PTC_) on the 100-nm nanoparticle when (b) *m* = 2 and (c) *m* = 4. Two and six stable trapping positions occur for *m* = 2 and 4, respectively. (d) Comparison of the PTOF and *F*_all_ when *m* = 2. The two types of optical forces become equivalent at the ‘0’ point (maximum-intensity point), meaning that only the PTOF exhibits in the circle. Polarization angles *θ* are 45° and −45° for solid and dashed curves, respectively. (e) Dependence of the PTOF on the PTC. The maximum PTOF increases linearly with *m* due to the enhanced polarization variations when *m* increases.

For a linearly polarized lightwave in cylindrical coordinates, its electric field can be expressed as ${{\bf E}} = A( {\cos \theta {{{{\bf \hat{e}}}}}_\rho + \sin \theta {{{{\bf \hat{e}}}}}_\varphi } )\exp ( {i\omega t - ikz} )$, where *θ* denotes the polarization angle of the electric field, defined as the angle between the polarization and radial directions (Fig. 1c); *A* is the amplitude of the electric field; and *ω* and *k* are the frequency and wave number in the medium, respectively. The IPM, which is known as ${\mathop{\mathrm{Im}}\nolimits} ( {{\bf \Pi }} ) = \frac{1}{{2{c}^2}}{\mathop{\mathrm{Im}}\nolimits} ( {{{\bf E}} \times {{{\bf H}}}^*} )$, is proportional to the time-averaged imaginary Poynting vector $\frac{1}{2}{\mathop{\mathrm{Im}}\nolimits} ( {{{\bf E}} \times {{{\bf H}}}^*} )$. It can be divided into three parts (consider the component in the azimuthal direction; see Methods for details):


(1) \begin{eqnarray*}
{\left. {{\mathop{\mathrm{Im}}\nolimits} ({{\bf \Pi }})} \right|}_\varphi &=& \frac{1}{{2{c}^2}}{\mathop{\mathrm{Im}}\nolimits} \left( {{E}_z{H}_\rho ^ * - {E}_\rho {H}_z^ * } \right)\\
&=& {\mathop{\mathrm{Im}}\nolimits} {({{\bf \Pi }})}_{\nabla {\mathrm{|}}E{\mathrm{|}}} + {\mathop{\mathrm{Im}}\nolimits} {({{\bf \Pi }})}_{{\mathrm{PTC}}}\! +\! {\mathop{\mathrm{Im}}\nolimits} {({{\bf \Pi }})}_{{\mathrm{mix}}}, \\
\end{eqnarray*}

where Im(**Π**)_∇|_*_E_*_|_ is associated with the |*E*| asymmetry: ∂*A*/∂*ρ*, with *ρ* being the radial position of the electric field (Fig. 1c); Im(**Π**)_PTC_ is related to the polarization inhomogeneity: ∂*θ*/∂*φ*, where *φ* is the azimuthal angle; and Im(**Π**)_mix_ is the term arising from the combined effects of the polarization inhomogeneity and |*E*| or phase asymmetry. Furthermore, the IPM force can be decomposed into three components: the PTOF (*F*_PTC_), *F*_∇|_*_E_*_|_, and *F*_mix_, which are, respectively, dependent on Im(**Π**)_PTC_, Im(**Π**)_∇|_*_E_*_|_, and Im(**Π**)_mix_. At the point of maximum electric field with a symmetric distribution (Fig. 1a), both Im(**Π**)_∇|_*_E_*_|_ and Im(**Π**)_mix_ vanish, leaving only Im(**Π**)_PTC_. Therefore, placing the particle at those positions is a feasible way to investigate the PTOF. The PTC, tied to the winding number of polarizations, strongly correlates with the polarization gradient: $m = \partial \theta /\partial \varphi + 1$. Eventually, the PTOF has a linear relationship with *m*, which is discussed in Fig. 2 and the Methods section.

Fundamentally, polarization inhomogeneity occurs ubiquitously, as a vector beam after focusing can induce this effect by the spin–orbit interaction (Fig. 1a). To rule out the influence of the intensity-gradient IPM force, we generate a doughnut-shaped light beam by passing the linearly polarized laser (532 nm) through a vortex half-wave plate and investigate the PTOF on particles placed at the intensity center (Fig. 1c). The electric field of the lightwave can be expressed as


(2) \begin{eqnarray*}
{{\bf E}} &=& {A}_E\left\{ {\cos \left[ {{\theta }_0 + \left( {m - 1} \right)\varphi } \right]{{{{\bf \hat{e}}}}}_\rho} \right.\\
&&\left. { + \sin \left[ {{\theta }_0 + \left( {m - 1} \right)\varphi } \right]{{{{\bf \hat{e}}}}}_\varphi } \right\}\\
&&\times \exp\! \left[ { - {{\left( {\frac{{\rho - {r}_s}}{w}} \right)}}^2} \right]\\
&&\times \exp \left[ {i\left( {\omega t - kz + \phi } \right)} \right] + {E}_z{{{\bf \hat{e}}}}_z,
\end{eqnarray*}

where *A_E_* is the amplitude; *θ*_0_ refers to the polarization angle at *φ* = 0°; *ρ* is the radial position of the electric field (Fig. 1c); *ϕ* refers to phase at *z* = 0 and *t* = 0; *r*_s_ and *w* denote the radius and the half width of the ring, respectively; ${{{\bf \hat{e}}}}_\rho $ and ${{{\bf \hat{e}}}}_\varphi $ are electric fields in radial and azimuthal directions, respectively; and *E_z_* represents the component of **E** in the *z* direction. The optical beam is simplified to approximately parallel propagation, neglecting variations of beam waists. The simulation results under practical experimental conditions align well with those derived from the simplified model, as shown in Figs S7 and S8.

Polarizations in this lightwave exhibit a spatial distribution across different azimuthal angles *φ*, with their patterns becoming increasingly varied as the PTC changes, as illustrated in Fig. 1b. The polarization angle rotates by *m* × 360° in space for a PTC of *m*. However, because the radial direction itself completes a full 360° rotation over one circle, the resulting polarization angle *θ* at a given *m* is given by (*m* − 1) × 360°. In linearly polarized gradient optical fields with symmetric intensity, the PTOF emerges as the predominant optical force, substantially exceeding other contributions such as those arising from spin momentum and spin–orbit interaction, as demonstrated in Fig. S6a. Under our experimental conditions, the PTOF is determined solely by the polarization state and its spatial gradient. Owing to the inherent periodicity of the polarization distribution, the resulting PTOF exhibits a corresponding periodic structure. Given that the topological charge of *θ* is *m* − 1, and the PTOF exhibits a 180° periodicity in *θ*, the number of periods for the PTOF is twice the topological charge of *θ*, equaling 2(*m* − 1). Radial light focusing generates an optical gradient force that confines the particle along the circle of maximum light intensity (hereafter simply called ‘the circle’). This approach establishes a framework for studying PTOF by eliminating contributions from |*E*|-asymmetry forces due to the IPM and spin angular momentum [46]. Force distributions for different PTCs are shown in Fig. 1c. We define positive and negative optical forces as those acting in counter clockwise and clockwise directions, respectively. The optical force remains constant along the circle, enabling continuous particle rotation at *m* = 1. The rotation direction can be clockwise or counter clockwise, depending on the fixed negative (*θ* < 0°) and positive (*θ* > 0°) polarization angles, respectively (see Fig. S2). The polarization angle starts to vary profoundly along the circle at high-order topologies (*m* > 1). This variation gives rise to the special distribution of positive and negative PTOFs, allowing particles to be trapped in potential wells [25,39]. PTOFs create two, four, and six force-equilibrium positions and an equal number of potential wells for *m* = 2, 3 and 4, respectively, as shown in Fig. 1c and e. The number of trapping positions is given by 2(*m* − 1), as determined by the winding number of polarizations. These potential wells are uniformly distributed along the intensity ring, with equal spacing between adjacent wells.

To elucidate the mechanism of the PTOF, we derive the theoretical expression for the optical force in the doughnut-shaped light beam. Our theoretical analysis (see Note S2) reveals that the PTOF depends both on the PTC and the polarization angle, which can be expressed as


(3) \begin{eqnarray*}
\left. F \right|_{\rho \,\, = \,\,{r}_s}^\varphi &=& \frac{{\omega {k}^3}}{{12 \pi }}{\mathrm{Im}}\left( {{\alpha }_{\rm ee}\alpha _{\rm mm}^{\mathrm{*}}} \right)\left. {{\mathrm{Im}}\left( {{{\bf E}} \times {{{\bf H}}}^{\mathrm{*}}} \right)} \right|_{\rho \,\, = \,\,{r}_s}^\varphi \\
&=& \frac{{{A}_E^2{k}^3m}}{{24\pi \mu {\mu }_0{r}_s}}{\mathrm{Im}}\left( {{\alpha }_{\rm ee}\alpha _{\rm mm}^{\mathrm{*}}} \right)\left( {2 + \frac{{{m}^2 - m}}{{{k}^2{r}_s^2}}} \right)\\
&&\times \sin \left[ {2{\theta }_0 + 2\left( {m - 1} \right)\varphi } \right],
\end{eqnarray*}

where *α*_ee_ and *α*_mm_ are electric and magnetic polarizabilities, respectively. As mentioned previously (also see Methods), for a relatively large *r_s_* satisfying $({m}^2 - m) \ll {k}^2{r}_s^2$ in this work, the maximum PTOF increases linearly with *m* and can be expressed as


(4) \begin{eqnarray*}
\left. F \right|_{\rho = {r}_s}^\varphi &=& \displaystyle\frac{{{A}_E^2{k}^3m}}{{12\pi \mu {\mu }_0{r}_s}}{\mathrm{Im}}\left( {{\alpha }_{\rm ee}\alpha _{\rm mm}^{\mathrm{*}}} \right)\\
&&\times \,\sin \left[ {2{\theta }_0 + 2\left( {m - 1} \right)\varphi } \right].
\end{eqnarray*}

While the PTOF increases with *m*, the potential-well depth |*U*| in two dimensions remains almost identical for various *m* because of the dominant conservative optical gradient force in the radial direction, as shown in Fig. 1e. Notably, |*U*| from the conservative PTOF can be expressed as


(5) \begin{eqnarray*}
\left| {{U}_{{\mathrm{PTC}}}} \right| &=& \left| { - \mathop \int \nolimits_L F\left( {\rho ,\varphi ,z} \right){\mathrm{d}}L} \right|\\
&=& \left| {\frac{{{A}_E^2{k}^3m}}{{12\pi \mu {\mu }_0\left( {m - 1} \right)}}{\mathrm{Im}}\left( {{\alpha }_{\rm ee}\alpha _{\rm mm}^*} \right)} \right|,
\end{eqnarray*}

which also does not increase with *m*. This is because, in the azimuthal direction along the circle, the increased number of potential wells shortens the integral distance of the PTOF, resulting in $| {{U}_{{\mathrm{PTC}}}} | \propto m/( {m - 1} )$. Based on this analysis, |*U*_PTC_| decreases with *m* and eventually becomes almost unchanged when *m* is large. The phenomena of forces and potential wells are also observed in the experiment.

The total optical force can display positive and negative values on two sides of the circle, as shown in Fig. 2a. This stems from the |*E*|-asymmetry-induced optical force, that is, the force from Im(**Π**)_∇|_*_E_*_|_, as |*E*| decays in opposite directions on both sides of the light beam [28,47]. PTOFs do not change sign under |*E*| asymmetry and display two (*m* = 2) and six (*m* = 4) stable trapping positions (black circles), as illustrated in Fig. 2b and c, respectively. The PTOF appears to decay with increasing distance from the circle (dashed line), driven by the declining intensity, while displaying periodic oscillations between positive and negative values within the circle when *m* > 1.

The derived force formula can be validated through rigorous simulations employing the Minkowski stress tensor, as demonstrated in Fig. 2d. It can be seen that the PTOF does not find its maximum value exactly at *x* = 0 (the ‘circle’ position) but at a point close to it. This occurs because the light intensity decay from *x* = 0 reduces the PTOF, while the shorter radial distance for *x* < 0 amplifies this force due to the reduced *r_s_* or the faster variation of the polarization in the azimuthal direction [Eq. (4)]. Notably, as *x* > 0, the intensity decreases while *r_s_* increases, leading to a faster force decay on the right side of *x* = 0 compared to the left.

It is worth noting that the general optical force from the intensity gradient by light focusing and spin–orbit interaction is negligible, as demonstrated in the force decomposition in Fig. S6. This facilitates the investigation of optical forces from the IPM. Both theory and numerical results show that only the PTOF is present at *x* = 0, as the symmetry of the electric field eliminates |*E*|-asymmetry-induced optical forces, as shown in Fig. 2d. The maximum PTOF increases linearly with the PTC due to the faster variation of the polarization angle at larger *m* (Fig. 2e). Additionally, both results demonstrate that this force is strongly correlated with the polarization angle, as explained by Eq. (4).

**Figure 3.**
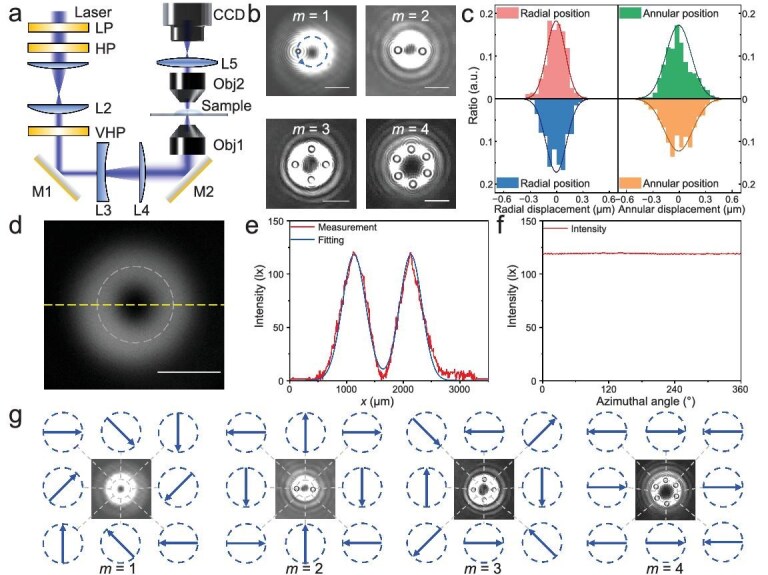
Experimental configurations. (a) Experimental setup. Laser, *λ* = 532 nm; LP, linear polarizer; HP, half-wave plate; VHP, vortex half-wave plate; M, mirror; L1, lens 1 (150 mm); L2, lens 2 (75 mm); L3, lens 3 (150 mm); L4, lens 4 (150 mm); L5, lens 5 (100 mm); Obj1, objective lens 1, 10×, NA = 0.25; and Obj2, objective lens 2, 40×, NA = 0.65. (b) Particle behaviors with various PTCs. Particles rotate counter clockwise when *m* = 1 and *θ* = 45°. 2(*m* − 1) particles can be trapped in potential wells when *m* > 1. Scale bars equal 10 μm. (c) Particle distributions demonstrate stable trapping in both radial and angular directions, measured under a laser intensity of ∼13.9 mW/μm^2^ with the topological charge *m* = 2. These distributions can be utilized for calculating optical forces. (d) Microscopic photograph of the vector beam. (e) Measured and fitted intensity profiles of the horizontal dashed line in (d). (f) Measured intensity profiles of the circular dashed line in (d). The uniform intensity along the circle agrees well with our design in Figs 1 and 2, showing negligible influence of the optical gradient force. (g) Measured polarization distributions at different *m*. The value of *θ* changes by (*m* − 1) × 360° over one full circle, while the polarization direction rotates *m* × 360° in space when the PTC is *m*.

Experimentally, the topological vector beam generated with a vortex half-wave plate is focused onto the sample plane, as shown in Fig. 3a. The PTC generates 2(*m* − 1) potential wells for particle trapping (Fig. 3b). Three-micrometer particles are confined within the circle of the vector beam by the optical gradient force acting in the radial direction (Fig. 3c). This confinement ensures that particles are solely influenced by the PTOF. The uniform intensity along the circle (Fig. 3d–f) agrees well with our design in Figs 1 and 2, showing negligible influence of the optical gradient force, also facilitating the observation of the PTOF.

Intriguingly, as obtained from Fig. 3c, the potential well depth of the radial optical gradient force is measured to be ∼839 *k*_B_*T*, while that of the annular/azimuthal PTOF reaches 1260 *k*_B_*T* (under conditions of *m* = 2 and laser intensity 13.9 mW/μm^2^). The maximum PTOF is measured to be 0.898 pN, which is approximately half of the maximum gradient force (2.16 pN), indicating the notable trapping capability of the PTOF. The large PTOF enables versatile manipulation, including rotation, trapping, and rotational particle array. Notably, considering the condition *r_s_* > *w* [Eq. (2)], while the maximum PTOF remains smaller than the optical gradient force, the potential well depth of the PTOF may exceed that of the optical gradient force due to the longer integration path (see Methods). Measured polarization states along the circle when *m* = 1, 2, 3, and 4 are presented in Fig. 3g, indicating that the polarization rotates *m* × 360° in one circle when the PTC is *m*.

**Figure 4.**
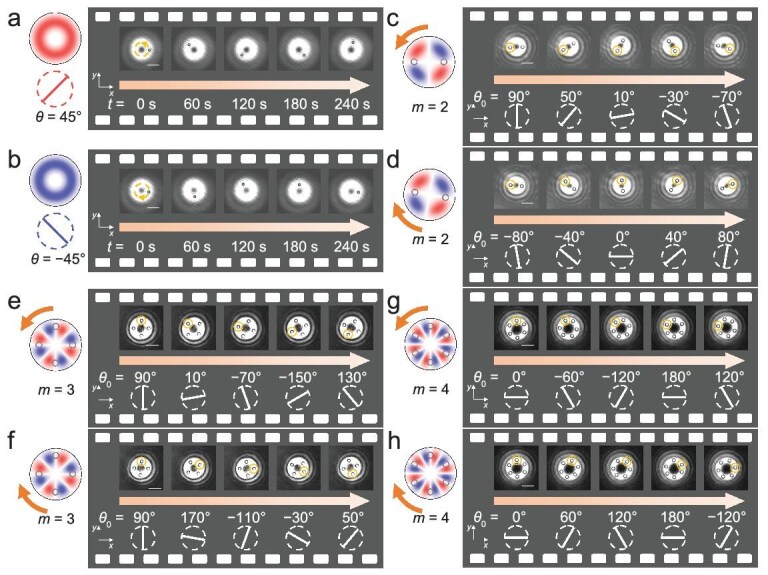
Experimental visualization of the high-order topology in the IPM via particle dynamics. Three-micrometer particles rotate (a) counter clockwise and (b) clockwise when *θ* = 45° and −45°, respectively, at *m* = 1. (c and d) Rotation of a particle pair when *m* = 2. (e and f) Rotation of a four-particle array when *m* = 3. (g and h) Rotation of a six-particle array when *m* = 4. The particle array can rotate clockwise and counter clockwise depending on different variations of the polarization angle at *φ* = 0°. Scale bars equal 10 μm. (a–h) can also be found in Supplementary Movies S1–S8, respectively.

Experimental observations demonstrate that particles rotate in the counter clockwise direction at *θ* = 45° (Fig. 4a) and in the clockwise direction at *θ* = −45° (Fig. 4b). Additionally, two, four, and six potential wells are observed for particle trapping when *m* = 2, 3, and 4, respectively (Fig. 4c–h), which align well with the theoretical predictions in Figs 1 and 2. The potential-well array containing a variable number of particles can be rotated clockwise or counter clockwise by altering the polarization angle using the half-wave plate. Particles are uniformly distributed along the circle, maintaining equal spacing between each other and moving with identical velocities.

**Figure 5.**
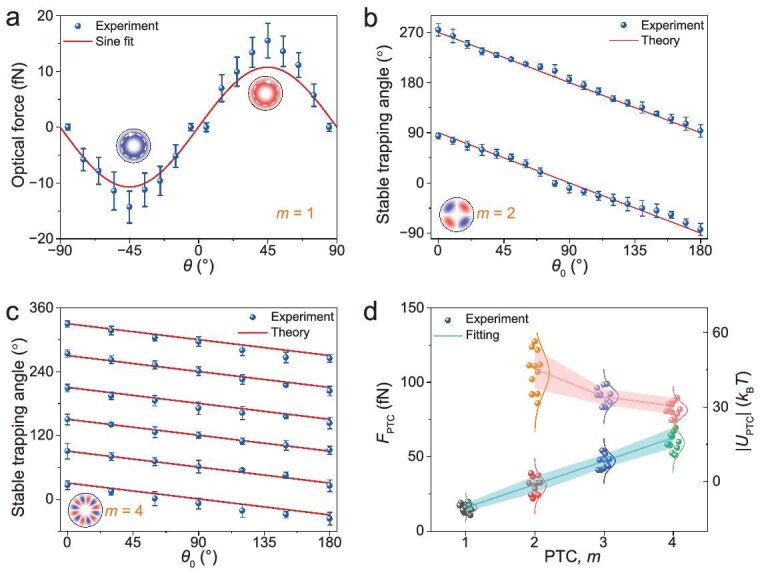
Experimental measurement of the PTOF by the IPM. (a) The optical force exhibits a sinusoidal dependence on the polarization angle *θ* when *m* = 1. (b) Two and (c) six stable trapping angles emerge at a given *θ*_0_ when *m* = 2 and 4, respectively. (d) Measured optical forces (*θ*_0_ = 45°) and potential wells as a function of the PTC. (a−d) are measured under a laser intensity of ∼0.79 mW/μm^2^, with each data point derived from 12 independent measurements. The fitting curves are obtained by applying Eqs (4) and (5) to the experimental data. The potential-well depth decreases slowly with the increasing PTC because the integral path for the optical force becomes shorter at larger *m*.

Figure 5a plots measured optical forces as a function of *θ* when *m* = 1. Optical forces are measured using the fluidic drag force, which can be expressed as *F*_drag_ = 6$\pi$*ηrv*, where *η* = 0.899 × 10^–3^ Pa s is the viscosity of the liquid at the temperature of 298 K [7,48]; *v* and *r* are the velocity and radius of the particle, respectively. The optical force exhibits a sinusoidal dependence on the polarization angle *θ*, with a period of 180°, as predicted in Eq. (4). Stable angles for trapping change linearly with *θ*_0_ when *m* > 1, as shown in Fig. 5b and c. This indicates that the potential-well array can be rotated by adjusting the polarization angle *θ*_0_ via mechanical rotation the half-wave plate to several specific angles, as demonstrated in Fig. 4. There are two, four, and six stable trapping positions corresponding to *m* = 2, 3, and 4, respectively, as revealed in simulation results in Figs 2, S3, and S4. When *θ*_0_ is varied by Δ*θ* via mechanical rotation of a half-wave plate, the angular coordinate of each stable trapping position shifts by −Δ*θ*, −Δ*θ*/2, and −Δ*θ*/3, respectively. Based on these simulation results, we plot the trajectories of each stable trapping position as a function of *θ*_0_. The measured PTOF and potential-well depth by the PTOF (|*U*_PTC_|) at different *m* are shown in Fig. 5d. The maximum PTOF increases linearly with *m*, as indicated in Fig. 2e and Eq. (4). However, the integral length (*L*) for $| {{U}_{{\mathrm{PTC}}}} | = \smallint {F}_{{\mathrm{PTC}}}{\mathrm{d}}L$ exhibits a relationship of 1/[2(*m* − 1)] with *m*, where *m* is the PTC. Therefore, the potential-well depth decreases with the PTC and remains nearly constant for larger values of *m* as $| {{U}_{{\mathrm{PTC}}}} | \propto m/( {m - 1} )$. These measurements validate the effectiveness of our method in probing intriguing PTOFs.

## DISCUSSION

It is important to note that the number of potential wells is not limited to six, as seen in the case of *m* = 4. It increases linearly with the PTC, following the relationship 2(*m* − 1). Future exploration of this PTOF can be pursued by configuring two-dimensional or even three-dimensional polarization-variation vector fields, enabling the movement and trapping of particles with a high degree of freedom. Meanwhile, approaches that increase the variation of the polarization state or the intensity of the light field can both enhance this intriguing PTOF, for instance, by optimizing the distribution of the polarization topology in the optical field, increasing the topological charge of the vector beam, increasing the intensity, or reducing the focusing range of the beam. Another effective method to enhance the PTOF is to use materials with higher refractive indices or absorbance. It is worth noting that the PTOF on the gold nanoparticle is two orders of magnitude greater than that on the polystyrene nanoparticle with an identical size. This is due to the complex refractive index of gold nanoparticle that significantly enhances the PTOF (Fig. S5). Except for the magnitude, the distribution pattern of the PTOF on the gold particle is the same as the polystyrene particle.

The PTOF represents a prevalent phenomenon in optical beams. As illustrated in Fig. 1a, optical beam convergence typically generates a polarization gradient, which gives rise to the PTOF. Moreover, vector beams intrinsically exhibit polarization gradients and thus inherently support the existence of the PTOF, such as azimuthally polarized beams and Lissajous vector beams. As a result, the PTOF is commonly observed across a wide variety of optical beams. However, the PTOF frequently coexists with optical gradient forces and spin-momentum-induced optical forces, rendering its observation and measurement highly challenging. To effectively isolate and quantify the PTOF, we employ highly symmetric optical fields, which enhance the dominance of the PTOF within the total optical force while minimizing the influence of other forces. The total optical force is calculated using the Minkowski stress tensor via Eq. (18) in Methods. For general vector beams, the expression for the PTOF is derived as


(6) \begin{eqnarray*}
{\left. {{F}_{{\mathrm{PTC}}}} \right|}_\varphi &=& \frac{{\omega {c}^2{k}^3}}{{6\pi }}{\mathrm{Im}}\left( {{\alpha }_{ee}\alpha _{mm}^{\mathrm{*}}} \right){\left. {{\mathop{\mathrm{Im}}\nolimits} {{({{\bf \Pi }})}}_{{\mathrm{PTC}}}} \right|}_\varphi \\
&=& \frac{k}{{12\pi \mu {\mu }_0{\rho }^3}}{\mathrm{Im}}\left( {{\alpha }_{ee}\alpha _{mm}^{\mathrm{*}}} \right)\\
&&\times \left\{ {{k}^2{\rho }^2\left[ {2{E}_\rho {E}_\varphi ^ * + \frac{{\partial \theta }}{{\partial \varphi }}\left( {{E}_\varphi ^ * \frac{{\partial {E}_\varphi }}{{\partial \theta }} - {E}_\rho \frac{{\partial {E}_\rho ^ * }}{{\partial \theta }}} \right)} \right]} \right.\\
&&\left. - \left( {{E}_\rho + \frac{{\partial {E}_\varphi }}{{\partial \theta }}\frac{{\partial \theta }}{{\partial \varphi }}} \right)\left[ {\frac{{\partial \theta }}{{\partial \varphi }}\left( {\frac{{\partial {E}_\rho ^ * }}{{\partial \theta }} + \frac{{{\partial }^2{E}_\varphi ^ * }}{{\partial {\theta }^2}}\frac{{\partial \theta }}{{\partial \varphi }}} \right)} \right.\right. \\
&&\left.\left. {\qquad \qquad \qquad\qquad +\, \frac{{\partial {E}_\varphi ^ * }}{{\partial \theta }}\frac{{{\partial }^2\theta }}{{\partial {\varphi }^2}}} \right] \right\}.
\end{eqnarray*}

The PTOF depends on polarization gradients of optical beams. In contrast to optical forces depending on intensity gradient [49,50], the PTOF can persist in regions characterized by uniform or symmetric intensity distributions. Under conditions of inhomogeneous intensity, the intensity-dependent forces are evaluated using ${F}_{\nabla | E |} = \frac{{\omega {c}^2{k}^3}}{{6\pi }}{\mathrm{Im}}( {{\alpha }_{\rm ee}\alpha _{\rm mm}^{\mathrm{*}}} ){\mathop{\mathrm{Im}}\nolimits} {({{\bf \Pi }})}_{\nabla | E |}$ and ${F}_{{\mathrm{mix}}} = \frac{{\omega {c}^2{k}^3}}{{6\pi }}{\mathrm{Im}}( {{\alpha }_{\rm ee}\alpha _{\rm mm}^{\mathrm{*}}} ){\mathop{\mathrm{Im}}\nolimits} {({{\bf \Pi }})}_{{\mathrm{mix}}}$, including Eqs (13) and (14) provided in Methods. Here, *F*_∇|_*_E_*_|_ is exclusively associated with the intensity gradient, whereas *F*_mix_ arises from both the polarization gradient and the intensity gradient. Notably, both forces vanish in regions of uniform or symmetrically distributed intensity, as shown in Figs S9 and S10.

Our work introduces a novel paradigm for constructing potential-well arrays solely from polarization angle distributions, without relying on conventional forces such as optical gradient forces, which are typically employed in multi-trapping techniques such as holographic optical tweezers [51–55], diffractive optical elements [56,57], acousto-optic modulators [58], wave interference [47], metasurfaces [59], nonlinearity [60,61], and so forth. Beyond trapping arrays, the PTOF endows another degree of freedom in optical manipulation, that is, rotation. Various numbers of particles can rotate individually or in an array given different PTCs.

Our study unravels the mystery of the IPM through a holistic theoretical framework and demonstrates its intrinsic high-order topological characteristics, which give rise to the intriguing PTOF. Generally, light fields with spatial distributions of polarization states can generate this IPM-induced PTOF (see Methods). This mechanism distinguishes itself from the previously studied forces in the IPM, which originate from the |*E*| asymmetry. The PTOF is typically conjugated with various forces, such as intensity or phase gradient forces, in many light fields. To isolate and observe the PTOF, it is feasible to employ a symmetric electric field with a uniform phase profile. Different PTCs induce distinct particle dynamics. For *m* = 1, the particle undergoes continuous rotation—either clockwise or counter clockwise—with the direction dictated by the fixed polarization angle *θ* along the circle. In higher-order topological fields (*m* > 1), particles are trapped by an array of rotational potential wells. The trapping force and number of potential wells increase and decline with the PTC, respectively. It is noteworthy that topological properties of intensities in light beams with complex polarization distributions have been investigated previously [62]. Focused cylindrical vector beams with *m* = 1 produce a ring-shaped intensity relief, whereas those with *m* > 1 generate pits and antinodes, with the number of antinodes equaling 2(*m* − 1). However, only the topological property of the intensity distribution is discussed. In such cases, the pronounced intensity gradient gives rise to a substantial optical gradient force, which hinders the observation of forces associated with the IPM. In contrast, the intensity along the ring remains consistent, mitigating the influence from the optical gradient force and facilitating the observation of the PTOF, as shown in Fig. S7.

The origin of the PTOF lies not in the spatial distribution of intensity, phase, spin, or orbital momentum, but in the topological property of the polarization structure, which fundamentally distinguishes it from other optical forces. Optical gradient forces depend on intensity inhomogeneity [42]. Spin momentum forces arise from inhomogeneous spin angular momentum and could be observed in linearly shaped [46] or Gaussian-shaped [21,63] optical fields with circular polarization. Forces from orbital momentum are associated with energy flow [64,65], including the phase gradient force determined by the phase gradient [66]. Spin–orbit interaction describes the mutual conversion between spin momentum and orbital momentum, which can occur in tightly focused optical fields [67], near dielectric interfaces [68], or under conditions of asymmetric scattering (such as surface plasmon polaritons) [65]. Nevertheless, the associated optical forces still directly originate from spin momentum or orbital momentum. Chirality-dependent optical forces act on particles with specific chiral responses [41,43,44]. In experiments, the optical gradient force manifests as pushing particles toward regions of higher field intensity. Other optical forces lack a direct experimental criterion and thus require simulation and comparative analysis based on measured field parameters to ascertain their respective contributions within the specific experimental context, as demonstrated in Figs S6a and S8b.

It is also worth noting that the PTOF differs fundamentally from the widely reported optical forces produced by phase singularities with varying topological charges in vortex beams. We unveil the intrinsic connection between the PTOF and the IPM, suggesting a convincing way to visualize the IPM. This study opens new avenues for extensive future research in this emerging field, including the exploration of optical forces in diverse polarization topologies. It underscores the significant potential for applications in optical sorting, binding [69], and critical studies in biological and quantum physics.

## METHODS

### Analysis of the IPM

Considering a linearly polarized vector beam propagating along the +*z* direction, its electric field can be expressed as


(7) \begin{eqnarray*}
{{\bf E}} = \left( {{E}_\rho {{{{\bf \hat{e}}}}}_\rho + {E}_\varphi {{{{\bf \hat{e}}}}}_\varphi } \right) + {E}_z{{{\bf \hat{e}}}}_z,
\end{eqnarray*}

where *E_ρ_, E_φ_*, and *E_z_* represent the component of **E** in the *ρ, φ*, and *z* direction, respectively. *E_z_* will be calculated below according to Maxwell’s equations. We use *θ* and *A* to represent the polarization angle and the amplitude of the electric field, respectively, wherein $| {\sin \theta } | = | {{{{E}_\varphi } / A}} |$.

We consider ${E}_z = v( {\rho ,\varphi ,z} ) \exp [ {i}( {\omega} { t - kz} + {\phi } ) ]$ as a time-harmonic electromagnetic wave, where *ω* and *k* are the frequency, and the wave number of light in the medium, respectively; *ϕ* refers to phase at *z* = 0 and *t* = 0. $v( {\rho ,\varphi ,z} )$ is assumed to be a slowly varying function, that is, $| {\partial v/\partial z} | \ll | {ikv} |$. The optical beam is simplified to approximately parallel propagation. Then, we can get


(8) \begin{eqnarray*}
\frac{{\partial {E}_z}}{{\partial z}} = \left( {{v}^{ - 1}\frac{{\partial v}}{{\partial z}} - ik} \right){E}_z \simeq - ik{E}_z.
\end{eqnarray*}

On the other hand, electromagnetic waves are required to adhere to Maxwell’s equations, including $\nabla \cdot {{\bf D}} = {\rho }_f$, where **D** represents the electric displacement vector, and ${\rho }_f$ is the free charge density. By considering the conditions ${{\bf D}} = \varepsilon {\varepsilon }_0{{\bf E}}$ and ${\rho }_f = 0$, where *ε* represents the relative dielectric permittivity, it is reasonable to obtain $\nabla \cdot {{\bf E}} = 0$. By introducing ${E}_z$ as an undetermined term and substituting ${E}_\rho $ and ${E}_\varphi $ into the equation $\nabla \cdot {{\bf E}} = 0$, it yields


(9) \begin{eqnarray*}
{E}_z \simeq - \frac{1}{{ik}}\frac{{\partial {E}_z}}{{\partial z}} = \frac{1}{{ik\rho }}\left[ {\frac{{\partial \left( {\rho {E}_\rho } \right)}}{{\partial \rho }} + \frac{{\partial {E}_\varphi }}{{\partial \varphi }}} \right].
\end{eqnarray*}

According to the Maxwell’s equation $\nabla \times {{\bf E}} = - i\omega \mu {\mu }_0{{\bf H}}$, where *μ* represents the relative magnetic permeability, the magnetic field is given by


(10) \begin{eqnarray*}
{{\bf H}} &=& - \frac{1}{{\omega \mu {\mu }_0\rho }}\left\{ {\left( {k\rho {E}_\varphi - i\frac{\partial }{{\partial \varphi }}{E}_z} \right){{{{\bf \hat{e}}}}}_\rho - \left( {k\rho {E}_\rho - i\rho \frac{\partial }{{\partial \rho }}{E}_z} \right){{{{\bf \hat{e}}}}}_\varphi }\right.\\
&&\left.{ + i\left[ {\frac{{\partial {E}_\rho }}{{\partial \varphi }} - \frac{{\partial \left( {\rho {E}_\varphi } \right)}}{{\partial \rho }}} \right]{{{{\bf \hat{e}}}}}_z} \right\} \\
&=&- \frac{1}{{\omega \mu {\mu }_0\rho }} \left\{ {\left\{ {k\rho {E}_\varphi - \frac{1}{{k\rho }}\frac{\partial }{{\partial \varphi }}\left[ {\frac{{\partial \left( {\rho {E}_\rho } \right)}}{{\partial \rho }} + \frac{{\partial {E}_\varphi }}{{\partial \varphi }}} \right]} \right\}{{{{\bf \hat{e}}}}}_\rho } \right.
&&\left. { - \left\{ {k\rho {E}_\rho - \frac{1}{k}\frac{\partial }{{\partial \rho }}\left[ {\frac{{\partial \left( {\rho {E}_\rho } \right)}}{{\partial \rho }} + \frac{{\partial {E}_\varphi }}{{\partial \varphi }}} \right]} \right.} \right.\\
&&\left. {\left. { + \frac{1}{{k\rho }}\left[ {\frac{{\partial \left( {\rho {E}_\rho } \right)}}{{\partial \rho }} + \frac{{\partial {E}_\varphi }}{{\partial \varphi }}} \right]} \right\}{{{{\bf \hat{e}}}}}_\varphi
} {+ i\left[ {\frac{{\partial {E}_\rho }}{{\partial \varphi }} \!-\! \frac{{\partial \left( {\rho {E}_\varphi } \right)}}{{\partial \rho }}} \right]{{{{\bf \hat{e}}}}}_z} \right\}\!. \\
\end{eqnarray*}

For simplicity, we assume that the value of *ϕ* and *A* are constant in the *φ* direction, while the value of *θ* is constant in the *ρ* direction (see Figs 1c and S7a). That is, $\frac{{\partial \phi }}{{\partial \varphi }} = 0$, $\frac{{\partial A}}{{\partial \varphi }} = 0$, and $\frac{{\partial \theta }}{{\partial \rho }} = 0$. In this case, the IPM in the *φ* direction can be expressed as


(11) \begin{eqnarray*}
{\left. {{\mathop{\mathrm{Im}}\nolimits} ({{\bf \Pi }})} \right|}_\varphi &=& \frac{1}{{2{c}^2}}{\mathop{\mathrm{Im}}\nolimits} \left( {{E}_z{H}_\rho ^ * - {E}_\rho {H}_z^ * } \right)\\
&=& \frac{1}{{2{c}^2k\omega \mu {\mu }_0{\rho }^2}}{\mathop{\mathrm{Re}}\nolimits} \left\{ {\left[ {\frac{{\partial \left( {\rho {E}_\rho } \right)}}{{\partial \rho }} + \frac{{\partial {E}_\varphi }}{{\partial \varphi }}} \right] }\right. \\
&&\times \left. {\left\{ {k\rho {E}_\varphi ^ * - \frac{1}{{k\rho }}\frac{\partial }{{\partial \varphi }}\left[ {\frac{{\partial \left( {\rho {E}_\rho ^ * } \right)}}{{\partial \rho }} + \frac{{\partial {E}_\varphi ^ * }}{{\partial \varphi }}} \right]} \right\}} \right\}\\
&&- \frac{1}{{2{c}^2\omega \mu {\mu }_0\rho }}{\mathop{\mathrm{Re}}\nolimits} \left\{ {{E}_\rho \left[ {\frac{{\partial {E}_\rho ^ * }}{{\partial \varphi }} - \frac{{\partial \left( {\rho {E}_\varphi ^ * } \right)}}{{\partial \rho }}} \right]} \right\}\\
&=& \frac{1}{{2{c}^2k\omega \mu {\mu }_0{\rho }^2}}\left\{ {\left( {{E}_\rho + \rho \frac{{\partial {E}_\rho }}{{\partial A}}\frac{{\partial A}}{{\partial \rho }} + \frac{{\partial {E}_\varphi }}{{\partial \theta }}\frac{{\partial \theta }}{{\partial \varphi }}} \right)}\right. \\
&&\times \left.{\left[ {k\rho {E}_\varphi ^ * - \frac{1}{{k\rho }}\frac{\partial }{{\partial \varphi }}\left( {\rho \frac{{\partial {E}_\rho ^ * }}{{\partial A}}\frac{{\partial A}}{{\partial \rho }} + {E}_\rho ^ * } \right.} \right.} \right. \\
&& + \frac{{\partial {E}_\varphi ^ * }}{{\partial \theta }}
\frac{{\partial \theta }}{{\partial \varphi }} \bigg)\! \bigg]\! +\! k\rho {E}_\rho \bigg( {{E}_\varphi ^ * \!+\! \rho \frac{{\partial {E}_\varphi ^ * }}{{\partial A}}\frac{{\partial A}}{{\partial \rho }} - \frac{{\partial {E}_\rho ^ * }}{{\partial \theta }}\frac{{\partial \theta }}{{\partial \varphi }}} \bigg) \\
&& - \frac{\rho }{k}\frac{{\partial {E}_\rho }}{{\partial \phi }}\frac{{{\partial }^2{E}_\rho ^ * }}{{\partial \theta \partial \phi }}{{\bigg( {\frac{{\partial \phi }}{{\partial \rho }}} \bigg)}}^2\frac{{\partial \theta }}{{\partial \varphi }} \big\}.
\end{eqnarray*}

Separating Eq. (11) into items only related to *θ* and others, we get


(12) \begin{eqnarray*}
{ {{\mathop{\mathrm{Im}}\nolimits} {{({{\bf \Pi }})}}_{{\mathrm{PTC}}}}
\big|}_\varphi &=& \frac{1}{{2{c}^2{k}^2\omega \mu {\mu }_0{\rho }^3}}\\
&&\bigg\{ {{k}^2{\rho }^2\bigg[ {2{E}_\rho {E}_\varphi ^ * + \frac{{\partial \theta }}{{\partial \varphi }}
\bigg( {{E}_\varphi ^ * \frac{{\partial {E}_\varphi }}{{\partial \theta }} - {E}_\rho \frac{{\partial {E}_\rho ^ * }}{{\partial \theta }}} \bigg)} \bigg]}\\
&& - \bigg( {{E}_\rho + \frac{{\partial {E}_\varphi }}{{\partial \theta }}\frac{{\partial \theta }}{{\partial \varphi }}} \bigg)\bigg[ {\frac{{\partial \theta }}{{\partial \varphi }}\bigg( {\frac{{\partial {E}_\rho ^ * }}{{\partial \theta }} + \frac{{{\partial }^2{E}_\varphi ^ * }}{{\partial {\theta }^2}}\frac{{\partial \theta }}{{\partial \varphi }}} \bigg)} \\
&&\qquad\qquad\qquad\qquad { + \frac{{\partial {E}_\varphi ^ * }}{{\partial \theta }}\frac{{{\partial }^2\theta }}{{\partial {\varphi }^2}}} \bigg] \bigg\},
\end{eqnarray*}

(13) \begin{eqnarray*}
{\left. {{\mathop{\mathrm{Im}}\nolimits} {{({{\bf \Pi }})}}_{\nabla \left| E \right|}} \right|}_\varphi = \frac{1}{{2{c}^2\omega \mu {\mu }_0}}\frac{{\partial A}}{{\partial \rho }}\left( {{E}_\rho \frac{{\partial {E}_\varphi ^ * }}{{\partial A}} + {E}_\varphi ^ * \frac{{\partial {E}_\rho }}{{\partial A}}} \right),
\end{eqnarray*}

(14) \begin{eqnarray*}
{ {{\mathop{\mathrm{Im}}\nolimits} {{({{\bf \Pi }})}}_{{\mathrm{mix}}}} \big|}_\varphi &=& - \frac{1}{{2{c}^2{k}^2\omega \mu {\mu }_0{\rho }^2}}\bigg\{ {\bigg( {{E}_\rho \!+\! \frac{{\partial {E}_\varphi }}{{\partial \theta }}\frac{{\partial \theta }}{{\partial \varphi }}} \bigg)\frac{{{\partial }^2{E}_\rho ^ * }}{{\partial A\partial \theta }}\frac{{\partial A}}{{\partial \rho }}\frac{{\partial \theta }}{{\partial \varphi }} }\\
&& { + \rho \frac{{\partial {E}_\rho }}{{\partial \phi }}\frac{{{\partial }^2{E}_\rho ^ * }}{{\partial \theta \partial \phi }}{{\bigg( {\frac{{\partial \phi }}{{\partial \rho }}} \bigg)}}^2\frac{{\partial \theta }}{{\partial \varphi }}} \\
&& + \frac{{\partial {E}_\rho }}{{\partial A}}\frac{{\partial A}}{{\partial \rho }} \bigg[ {\rho \frac{{{\partial }^2{E}_\rho ^ * }}{{\partial A\partial \theta }}\frac{{\partial A}}{{\partial \rho }}\frac{{\partial \theta }}{{\partial \varphi }} + \frac{{\partial {E}_\varphi ^ * }}{{\partial \theta }}\frac{{{\partial }^2\theta }}{{\partial {\varphi }^2}} + \frac{{\partial \theta }}{{\partial \varphi }} }
&&\quad\quad\quad\quad {\bigg( {\frac{{\partial {E}_\rho ^ * }}{{\partial \theta }} + \frac{{{\partial }^2{E}_\varphi ^ * }}{{\partial {\theta }^2}}\frac{{\partial \theta }}{{\partial \varphi }}} \bigg)} \bigg] \bigg\}.
\end{eqnarray*}

Here, Im(**Π**)_∇|_*_E_*_|_ is associated with the |*E*| asymmetry; Im(**Π**)_PTC_ is related to the polarization inhomogeneity; and Im(**Π**)_mix_ is the term arising from the combined effects of |*E*| or phase asymmetry and the polarization inhomogeneity.

When $\frac{{\partial A}}{{\partial \rho }} = 0$ and $\frac{{\partial \phi }}{{\partial \rho }} \ll k$ are satisfied at the same time, for instance, in positions with symmetric electric fields, we get ${ {{\mathop{\mathrm{Im}}\nolimits} ({{\bf \Pi }})} |}_\varphi = { {{\mathop{\mathrm{Im}}\nolimits} {{({{\bf \Pi }})}}_{{\mathrm{PTC}}}} |}_\varphi $. In other words, the IPM is completely determined by the PTC term.

The PTC (*m*), which describes the relationship between the polarization angle *θ* and the polar angle *φ*, can be expressed as


(15) \begin{eqnarray*}
m = \frac{{\partial \theta }}{{\partial \varphi }} + 1.
\end{eqnarray*}

Considering the condition when the PTC is very large and satisfies $({m}^2 - m) \gg {k}^2{\rho }^2$, the IPM becomes


(16) \begin{eqnarray*}
{ {{\mathrm{Im}}{{({\bf \Pi})}}_{{\mathrm{PTC}}}} \big|}_\varphi
&\simeq & - \frac{1}{{2{c}^2{k}^2\omega \mu {\mu }_0{\rho }^3}}\frac{{\partial \theta }}{{\partial \varphi }}\bigg( {{E}_\rho + \frac{{\partial {E}_\varphi }}{{\partial \theta }}\frac{{\partial \theta }}{{\partial \varphi }}} \bigg) \\
&&\times \bigg( {\frac{{\partial {E}_\rho ^*}}{{\partial \theta }} + \frac{{{\partial }^2{E}_\varphi ^*}}{{\partial {\theta }^2}}\frac{{\partial \theta }}{{\partial \varphi }}} \bigg) \\
&\propto& {m}^2 ( {m - 1}).\\
\end{eqnarray*}

In contrast, when the PTC is relatively small, that is, $({m}^2 - m) \ll {k}^2{\rho }^2$, we get


(17) \begin{eqnarray*}
{ {{\mathrm{Im}}{{({\bf \Pi})}}_{{\mathrm{PTC}}}} \big|}_\varphi &\simeq & \frac{1}{{2{c}^2\omega \mu {\mu }_0\rho }}\bigg[ {2{E}_\rho {E}_\varphi ^* + \frac{{\partial \theta }}{{\partial \varphi }} }\\
&& {\bigg( {{E}_\varphi ^*\frac{{\partial {E}_\varphi }}{{\partial \theta }} - {E}_\rho \frac{{\partial {E}_\rho ^*}}{{\partial \theta }}} \bigg)} \bigg] \\
& \propto& m.\\
\end{eqnarray*}

The radii of rings in the simulation and experiment are 6 and 5.75 μm, respectively. In the two cases, *k*^2^*ρ*^2^ ∼ 8800, which are much greater than *m*^2^ – *m*, meaning that the PTOF from Im(**Π**)_PTC_ should be proportional to *m*. This linear dependence of the PTOF on *m* has been confirmed in theory (Fig. 2e) and experiment (Fig. 5d).

### Simulation of the PTOF

The optical force can be modeled using the dipole and multipole theories (see details in Notes S2 and S3). The rigorous simulation of optical forces can be conducted using the Minkowski stress tensor, which can be expressed as [25,26]


(18) \begin{eqnarray*}
\left\langle {\bf F} \right\rangle = \oint_S
{\langle \displaystyle\mathop{{\bf T}}^{\leftrightarrow}\rangle } \cdot {\bf \hat{ n}}{\mathrm{d}\it S}
,\end{eqnarray*}

(19) \begin{eqnarray*}
\left\langle {{\bf T}_{ij}} \right\rangle = \frac{1}{2}\left[ {{D}_iE_j^* + {B}_iH_j^* - \frac{1}{2}\left( {{{\bf D}} \cdot {{{\bf E}}}^* + {{\bf B}} \cdot {{{\bf H}}}^*} \right){\delta }_{ij}} \right].
\end{eqnarray*}

Here, the symbol $\langle \cdot \rangle $ denotes time average, ${\bf \hat{n}}$ represents the unit outward normal to the integral surface, and ${\delta }_{ij}$ is Kronecker delta. The Minkowski stress tensor is directly coded in COMSOL and solved using Maxwell equations.

### Experimental configurations

To generate the doughnut-shaped vector beam with different PTCs, the 532-nm laser (Laser Quantum, mpc 6000) first passes through the linear polarizer and then the half-wave plate to control the linear polarization direction (Fig. 3a). Different vortex half-wave plates are utilized to generate the doughnut-shaped beam with various PTCs (Fig. S1), which is subsequently focused onto working plate. Three-micrometer polystyrene particles are diluted and dropped onto a slide on the working plate. Particle dynamics are observed via a CCD camera (JCOPTIX, AIC-501GM-GE) through a 40× objective lens.

The focused beam retains the polarization of the incident beam while acquiring a radial phase gradient, as shown in Fig. S7. Consequently, the PTOF emerges as the dominant force along the angular direction of the annular beam, as illustrated in Fig. S8, wherein optical forces arising from spin momentum and spin–orbit interaction vanish due to the symmetry of |*E*|.

### Analysis of the PTOF, the optical gradient force and their potential wells

We quantified the trapping stiffness of both the optical gradient force and the PTOF through analysis of the mean squared displacement (MSD) of Brownian motion. When the angular displacement to the equilibrium position *x_a_* is sufficiently small (that is, *x_a_* ≪ *r_s_*), the PTOF can be estimated according to Eq. (4) as


(20) \begin{eqnarray*}
{F}_{{\mathrm{PTC}}} &=& \frac{{( {m - 1} )}}{{{r}_s}}\big| {{U}_{{\mathrm{PTC}}}} \big|\sin \big[ {2{\theta }_0 + 2 ( {m - 1} )\varphi } \big] \\
& \simeq& \frac{{2 ( {m - 1})}}{{r_s^2}}\big| {{U}_{{\mathrm{PTC}}}} \big|{x}_a.
\end{eqnarray*}

The PTOF exhibits proportionality to the angular displacement, therefore, its trapping stiffness *k*_PTC_ can be derived as


(21) \begin{eqnarray*}
{k}_{{\mathrm{PTC}}} = \frac{{2{k}_{\mathrm{B}}T}}{{\left\langle {{x}_a^2} \right\rangle }},
\end{eqnarray*}

where *k*_B_ and *T* represent the Boltzmann constant and the temperature, respectively. And we can obtain the maximum PTOF $F_{{\mathrm{PTC}}}^{{\mathrm{max}}}$ by


(22) \begin{eqnarray*}
F_{{\mathrm{PTC}}}^{{\mathrm{max}}} = {k}_{{\mathrm{PTC}}}\frac{{{r}_s}}{2},
\end{eqnarray*}

and its potential well depth by


(23) \begin{eqnarray*}
\left| {{U}_{{\mathrm{PTC}}}} \right| = {k}_{{\mathrm{PTC}}}\frac{{{r}_s^2}}{{2\left( {m - 1} \right)}}.
\end{eqnarray*}

Similarly, the trapping stiffness ${k}_{{\mathrm{grad}}}$ can be approximated by MSD related to the radial displacement *x_r_* as


(24) \begin{eqnarray*}
{k}_{{\mathrm{grad}}} = \frac{{2{k}_{\mathrm{B}}T}}{{\left\langle {{x}_r^2} \right\rangle }}.
\end{eqnarray*}

For comparison, we can derive the maximum gradient force $F_{{\mathrm{grad}}}^{{\mathrm{max}}}$ as


(25) \begin{eqnarray*}
F_{{\mathrm{grad}}}^{{\mathrm{max}}} = {k}_{{\mathrm{grad}}}w,
\end{eqnarray*}

and its potential well depth $| {{U}_{{\mathrm{grad}}}} |$ as


(26) \begin{eqnarray*}
\left| {{U}_{{\mathrm{grad}}}} \right| = {k}_{{\mathrm{grad}}}\frac{{{w}^2}}{2}.
\end{eqnarray*}

The maximum PTOF and the maximum optical gradient force are proportional to *r_s_* and *w*, respectively, while the potential well depths of the PTOF and the optical gradient force are proportional to *r_s_*^2^ and *w*^2^, respectively.

## Supplementary Material

nwag171_Supplemental_Files
