# Supplementary material for: Imaginary Poynting momentum: polarization topology and versatile optical manipulation
Source: Natl Sci Rev. 2026 Mar 17;13(11):nwag171. doi: 10.1093/nsr/nwag171 (PMC13231513; doi:10.1093/nsr/nwag171)
Supplement: nwag171_Supplemental_Files [file nwag171_supplemental_files.zip › NSR_MS-2025-2420-SI-Yuchen Zhu.pdf]

Supplementary Materials for
Imaginary Poynting momentum: polarization topology and versatile optical manipulation

Yuchen Zhu[1, 2, 3, 4, †], Yuzhi Shi[1, 2, 3, 4, †, *], Tao He[1, 2, 3, 4, *], Qinghua Song[5], Weijin Chen[1, 2, 3, 4], Chengxing Lai[1, 2, 3, 4], Zeyong Wei[1, 2, 3, 4], Xiong Dun[1, 2, 3, 4], Zhanshan Wang[1, 2, 3, 4], C. T. Chan[6], Cheng-Wei Qiu[7, *] and Xinbin Cheng[1, 2, 3, 4, *]

[1] Institute of Precision Optical Engineering, School of Physics Science and Engineering, Tongji University, Shanghai 200092, China

[2] Technology Innovation Center of Mass Spectrometry for State Market Regulation, Center for Advanced Measurement Science, National Institute of Metrology, Beijing 100029, China

[3] MOE Key Laboratory of Advanced Micro-Structured Materials, Shanghai 200092, China

[4] Shanghai Institute of Intelligent Science and Technology, Tongji University, Shanghai 200092, China

[5] Tsinghua Shenzhen International Graduate School, Tsinghua University, Shenzhen 518055, China

[6] Department of Physics, The Hong Kong University of Science and Technology, Hong Kong 999077, China

[7] Department of Electrical and Computer Engineering, National University of Singapore, Singapore 117583, Singapore

*Corresponding authors. E-mails: yzshi@tongji.edu.cn; hetao@tongji.edu.cn; chengwei.qiu@nus.edu.sg; chengxb@tongji.edu.cn

†Equally contributed to this work.

Supplementary Note 1: Vortex half-wave plates and annular Gaussian beam

Vortex half-wave plates (VHPs) can be considered as half-wave plates with a continuously varying fast axis. The fast axis orientation (β) remains constant along the radial direction. However, β varies with the azimuthal angle (φ), as illustrated in Fig. S1. Specifically, in a VHP of order m, a full 360° rotation in φ corresponds to a change in β of $m \times 180°$. When a linearly polarized beam passes through the VHP, the output beam is also linearly polarized with polarization angle varying across different azimuthal angles. Since the incident laser light is a Gaussian beam, the outgoing light is approximately an annular Gaussian beam, as shown in Figs. 3d–f in the main text.

Supplementary Note 2: Derivation of the optical force in dipole approximation

To investigate the optical force from the imaginary Poynting momentum (IPM), the laser beam is focused by an objective lens into an annular focal spot with radius $r_s = 6$ μm and half width $w = 3$ μm, respectively. The electric field of the focused beam can be approximately written as

$$\mathbf{E} = A_E \left\{\cos\left[\theta_0 + (m-1)\varphi\right]\hat{\mathbf{e}}_\rho + \sin\left[\theta_0 + (m-1)\varphi\right]\hat{\mathbf{e}}_\varphi\right\}\exp\left[-\left(\frac{\rho - r_s}{w}\right)^2\right]\exp\left[i\left(\omega t - kz + \phi\right)\right] + E_z\hat{\mathbf{e}}_z, \quad \text{(S1)}$$

where A_E is the amplitude; φ is the azimuthal angle; where θ_0 refers to the polarization angle at $\varphi = 0°$; ρ is the position of the electric field; r_s is the radius of the ring; ϕ refers to phase at $z = 0$ and $t = 0$; E_ρ, E_φ and E_z represent the component of **E** in the ρ, φ, and z direction, respectively (see Fig. 1c); ω and k are the frequency and the wave number in the medium, respectively.

The optical beam is simplified to approximately parallel propagation, neglecting variations of beam waists. For simplicity, we assume that A and ϕ is constant in the φ direction and θ is constant in the ρ direction (see the illustration in Fig. 1c). That is, $\frac{\partial A}{\partial \varphi} = 0$, $\frac{\partial \phi}{\partial \varphi} = 0$, and $\frac{\partial \theta}{\partial \rho} = 0$. According to Maxwell's equations, we get the z-component of **E** and the magnetic field **H** as

$$E_z \simeq -\frac{1}{ik}\frac{\partial E_z}{\partial z} = \frac{1}{ik\rho}\left[\frac{\partial(\rho E_\rho)}{\partial \rho} + \frac{\partial E_\varphi}{\partial \varphi}\right], \quad \text{(S2)}$$

$$\begin{aligned}\mathbf{H}&=\frac{i}{\omega\mu\mu_0\rho}\left\{\left(\frac{\partial}{\partial\varphi}E_z-\rho\frac{\partial}{\partial z}E_\varphi\right)\hat{\mathbf{e}}_\rho+\left(\rho\frac{\partial}{\partial z}E_\rho-\rho\frac{\partial}{\partial\rho}E_z\right)\hat{\mathbf{e}}_\varphi+\left[\frac{\partial\left(\rho E_\varphi\right)}{\partial\rho}-\frac{\partial E_\rho}{\partial\varphi}\right]\hat{\mathbf{e}}_z\right\}\\&=-\frac{1}{\omega\mu\mu_0\rho}\left\{\left(k\rho E_\varphi-i\frac{\partial}{\partial\varphi}E_z\right)\hat{\mathbf{e}}_\rho-\left(k\rho E_\rho-i\rho\frac{\partial}{\partial\rho}E_z\right)\hat{\mathbf{e}}_\varphi+i\left[\frac{\partial E_\rho}{\partial\varphi}-\frac{\partial\left(\rho E_\varphi\right)}{\partial\rho}\right]\hat{\mathbf{e}}_z\right\}\\&=-\frac{1}{\omega\mu\mu_0\rho}\left\{\left\{k\rho E_\varphi-\frac{1}{k\rho}\frac{\partial}{\partial\varphi}\left[\frac{\partial\left(\rho E_\rho\right)}{\partial\rho}+\frac{\partial E_\varphi}{\partial\varphi}\right]\right\}\hat{\mathbf{e}}_\rho-\left\{k\rho E_\rho-\frac{1}{k}\frac{\partial}{\partial\rho}\left[\frac{\partial\left(\rho E_\rho\right)}{\partial\rho}+\frac{\partial E_\varphi}{\partial\varphi}\right]\right.\right.\\&\left.\left.\quad+\frac{1}{k\rho}\left[\frac{\partial\left(\rho E_\rho\right)}{\partial\rho}+\frac{\partial E_\varphi}{\partial\varphi}\right]\right\}\hat{\mathbf{e}}_\varphi+i\left[\frac{\partial E_\rho}{\partial\varphi}-\frac{\partial\left(\rho E_\varphi\right)}{\partial\rho}\right]\hat{\mathbf{e}}_z\right\}.\end{aligned}\tag{S3}$$

Substituting the background field, and neglecting the slow variation of phase, we can get

$$\begin{aligned}E_z&\simeq\frac{1}{ik\rho}\left[\frac{\partial\left(\rho E_\rho\right)}{\partial\rho}+\frac{\partial E_\varphi}{\partial\varphi}\right]\\&=\frac{A_E}{ik\rho}\left(m-2\rho\frac{\rho-r_s}{w^2}\right)\cos\left[\theta_0+\left(m-1\right)\varphi\right]\exp\left[-\left(\frac{\rho-r_s}{w}\right)^2\right]\exp\left(i\omega t-ikz\right)\\&=\frac{A_EK_E}{ik\rho}\cos\theta\exp\left[-\left(\frac{\rho-r_s}{w}\right)^2\right]\exp\left(i\omega t-ikz\right),\end{aligned}\tag{S4}$$

where $K_E=m-2\rho\frac{\rho-r_s}{w^2}$, and $\theta=\theta_0+\left(m-1\right)\varphi$, which are set for more concise expressions. And **H** can be expressed as

$$\begin{aligned}\mathbf{H}&=-\frac{1}{\omega\mu\mu_0\rho}\left\{\left(k\rho E_\varphi-i\frac{\partial}{\partial\varphi}E_z\right)\hat{\mathbf{e}}_\rho-\left(k\rho E_\rho-i\rho\frac{\partial}{\partial\rho}E_z\right)\hat{\mathbf{e}}_\varphi+i\left[\frac{\partial E_\rho}{\partial\varphi}-\frac{\partial\left(\rho E_\varphi\right)}{\partial\rho}\right]\hat{\mathbf{e}}_z\right\}\\&=-\frac{A_E}{\omega\mu\mu_0\rho}\left\{\left[k\rho+\frac{1}{k\rho}\left(m-1\right)\left(m-2\rho\frac{\rho-r_s}{w^2}\right)\right]\sin\left[\theta_0+\left(m-1\right)\varphi\right]\hat{\mathbf{e}}_\rho\right.\\&\quad-\left[k\rho+2\frac{\rho-r_s}{kw^2}\left(m-2\rho\frac{\rho-r_s}{w^2}\right)+\frac{1}{k\rho}\left(m+\frac{2\rho^2}{w^2}\right)\right]\cos\left[\theta_0+\left(m-1\right)\varphi\right]\hat{\mathbf{e}}_\varphi\\&\quad\left.-i\left(m-2\rho\frac{\rho-r_s}{w^2}\right)\sin\left[\theta_0+\left(m-1\right)\varphi\right]\hat{\mathbf{e}}_z\right\}\exp\left[-\left(\frac{\rho-r_s}{w}\right)^2\right]\exp\left(i\omega t-ikz\right)\\&=-\frac{A_E}{\omega\mu\mu_0\rho}\left\{\left[k\rho+\frac{1}{k\rho}\left(m-1\right)K_E\right]\sin\theta\hat{\mathbf{e}}_\rho-\left[k\rho+2\frac{\rho-r_s}{kw^2}K_E+\frac{1}{k\rho}\left(m+\frac{2\rho^2}{w^2}\right)\right]\cos\theta\hat{\mathbf{e}}_\varphi\right.\\&\quad\left.-iK_E\sin\theta\hat{\mathbf{e}}_z\right\}\exp\left[-\left(\frac{\rho-r_s}{w}\right)^2\right]\exp\left(i\omega t-ikz\right).\end{aligned}\tag{S5}$$

For an achiral spherical dipole embedded in a non-absorbing medium with relative permittivity ε and relative permeability μ, the electric and magnetic dipole moments of the sphere satisfy the following relationship:

$$\mathbf{p}=\alpha_{ee}\mathbf{E}, \tag{S6}$$

$$\mathbf{m}=\alpha_{mm}\mathbf{H}, \tag{S7}$$

where α_{ee} and α_{mm} denote electric and magnetic polarizabilities, respectively. The time-averaged electromagnetic force acting on an achiral dipole sphere is given as

$$\mathbf{F}=\frac{1}{2}\mathrm{Re}\left[\mathbf{p}\left(\nabla\otimes\mathbf{E}^*\right)\right]+\frac{1}{2}\mathrm{Re}\left[\mathbf{m}\left(\nabla\otimes\mathbf{H}^*\right)\right]-\frac{ck^4}{12\pi\sqrt{\varepsilon\mu}}\mathrm{Re}\left(\mathbf{p}\times\mathbf{m}^*\right). \tag{S8}$$

Substituting Eqs. (S6) and (S7) into Eq. (S8), we obtain

$$\mathbf{F}=\frac{1}{2}\mathrm{Re}\left[\alpha_{ee}\mathbf{E}\left(\nabla\otimes\mathbf{E}^*\right)+\alpha_{mm}\mathbf{H}\left(\nabla\otimes\mathbf{H}^*\right)-\frac{ck^4}{6\pi\sqrt{\varepsilon\mu}}\left(\alpha_{ee}\mathbf{E}\times\alpha_{mm}{}^*\mathbf{H}^*\right)\right]. \tag{S9}$$

We then adopt the following terms: $\mathbf{P}=\frac{1}{2}\mathrm{Re}\left(\mathbf{E}\times\mathbf{H}^*\right)$, representing the time-averaged Poynting vector, which is proportional to the time-averaged Poynting momentum density $\mathrm{Re}(\mathbf{\Pi})=\frac{1}{2c^2}\mathrm{Re}\left(\mathbf{E}\times\mathbf{H}^*\right)$; $\nabla\times\mathbf{L}_e=\frac{\varepsilon_0}{4\omega\mu i}\nabla\times\left(\mathbf{E}\times\mathbf{E}^*\right)$ and $\nabla\times\mathbf{L}_m=\frac{\mu_0}{4\omega\varepsilon i}\nabla\times\left(\mathbf{H}\times\mathbf{H}^*\right)$, denoting time-averaged spin angular momentum densities associated with electric and magnetic fields, respectively.

The first item on the right side of Eq. (S9) can be expressed as

$$\begin{aligned}
&\frac{1}{2}\mathrm{Re}\left[\alpha_{ee}\mathbf{E}\left(\nabla\otimes\mathbf{E}^*\right)\right]\\
&=\frac{1}{2}\mathrm{Re}\left[\alpha_{ee}\left(\mathbf{E}\cdot\nabla\right)\mathbf{E}^*+\alpha_{ee}\mathbf{E}\times\left(\nabla\times\mathbf{E}^*\right)\right]\\
&=\frac{1}{2}\mathrm{Re}\left(\alpha_{ee}\right)\mathrm{Re}\left[\left(\mathbf{E}\cdot\nabla\right)\mathbf{E}^*\right]+\frac{1}{2}\mathrm{Re}\left(\alpha_{ee}\right)\mathrm{Re}\left[\mathbf{E}\times\left(\nabla\times\mathbf{E}^*\right)\right]\\
&\quad-\frac{1}{2}\mathrm{Im}\left(\alpha_{ee}\right)\mathrm{Im}\left[\left(\mathbf{E}\cdot\nabla\right)\mathbf{E}^*\right]-\frac{1}{2}\mathrm{Im}\left(\alpha_{ee}\right)\mathrm{Im}\left[\mathbf{E}\times\left(\nabla\times\mathbf{E}^*\right)\right]\\
&=\frac{1}{4}\mathrm{Re}\left(\alpha_{ee}\right)\mathrm{Re}\left[\left(\mathbf{E}\cdot\nabla\right)\mathbf{E}^*+\mathbf{E}\times\left(\nabla\times\mathbf{E}^*\right)+\left(\mathbf{E}^*\cdot\nabla\right)\mathbf{E}+\mathbf{E}^*\times\left(\nabla\times\mathbf{E}\right)\right]\\
&\quad+\frac{1}{2}\mathrm{Im}\left(\alpha_{ee}\right)\mathrm{Re}\left[i\left(\mathbf{E}\cdot\nabla\right)\mathbf{E}^*\right]+\frac{1}{2}\omega\,\mathrm{Im}\left(\alpha_{ee}\right)\mathrm{Re}\left[\mathbf{E}\times\mathbf{B}^*\right]\\
&=\frac{1}{4}\mathrm{Re}\left(\alpha_{ee}\right)\nabla\left|\mathbf{E}\right|^2+\omega\mu\mu_0\,\mathrm{Im}\left(\alpha_{ee}\right)\mathbf{P}+\frac{\omega\mu}{\varepsilon_0}\mathrm{Im}\left(\alpha_{ee}\right)\nabla\times\mathbf{L}_e.
\end{aligned} \tag{S10}$$

Similarly, the second item on the right side of Eq. (S9) can be expressed as

$$\begin{aligned}
&\frac{1}{2}\mathrm{Re}\left[\alpha_{mm}\mathbf{H}\left(\nabla\otimes\mathbf{H}^*\right)\right] \\
&=\frac{1}{2}\mathrm{Re}\left[\alpha_{mm}\left(\mathbf{H}\cdot\nabla\right)\mathbf{H}^*+\alpha_{mm}\mathbf{H}\times\left(\nabla\times\mathbf{H}^*\right)\right] \\
&=\frac{1}{2}\mathrm{Re}\left(\alpha_{mm}\right)\mathrm{Re}\left[\left(\mathbf{H}\cdot\nabla\right)\mathbf{H}^*\right]+\frac{1}{2}\mathrm{Re}\left(\alpha_{mm}\right)\mathrm{Re}\left[\mathbf{H}\times\left(\nabla\times\mathbf{H}^*\right)\right] \\
&-\frac{1}{2}\mathrm{Im}\left(\alpha_{mm}\right)\mathrm{Im}\left[\left(\mathbf{H}\cdot\nabla\right)\mathbf{H}^*\right]-\frac{1}{2}\mathrm{Im}\left(\alpha_{mm}\right)\mathrm{Im}\left[\mathbf{H}\times\left(\nabla\times\mathbf{H}^*\right)\right] \\
&=\frac{1}{4}\mathrm{Re}\left(\alpha_{mm}\right)\mathrm{Re}\left[\left(\mathbf{H}\cdot\nabla\right)\mathbf{H}^*+\mathbf{H}\times\left(\nabla\times\mathbf{H}^*\right)+\left(\mathbf{H}^*\cdot\nabla\right)\mathbf{H}+\mathbf{H}^*\times\left(\nabla\times\mathbf{H}\right)\right] \\
&+\frac{1}{2}\mathrm{Im}\left(\alpha_{mm}\right)\mathrm{Re}\left[i\left(\mathbf{H}\cdot\nabla\right)\mathbf{H}^*\right]-\frac{1}{2}\omega\,\mathrm{Im}\left(\alpha_{mm}\right)\mathrm{Re}\left[\mathbf{H}\times\mathbf{D}^*\right] \\
&=\frac{1}{4}\mathrm{Re}\left(\alpha_{mm}\right)\nabla\left|\mathbf{H}\right|^2+\omega\varepsilon\varepsilon_0\,\mathrm{Im}\left(\alpha_{mm}\right)\mathbf{P}+\frac{\omega\varepsilon}{\mu_0}\mathrm{Im}\left(\alpha_{mm}\right)\nabla\times\mathbf{L}_m.
\end{aligned} \tag{S11}$$

And the last item on the right side of Eq. (S9) can be calculated as

$$\begin{aligned}
&\frac{1}{2}\mathrm{Re}\left[-\frac{ck^4}{6\pi\sqrt{\varepsilon\mu}}\left(\alpha_{ee}\mathbf{E}\times\alpha_{mm}{}^*\mathbf{H}^*\right)\right] \\
&=-\frac{ck^4}{6\pi\sqrt{\varepsilon\mu}}\mathrm{Re}\left(\alpha_{ee}\alpha_{mm}{}^*\right)\mathbf{P}+\frac{ck^4}{12\pi\sqrt{\varepsilon\mu}}\mathrm{Im}\left(\alpha_{ee}\alpha_{mm}{}^*\right)\mathrm{Im}\left(\mathbf{E}\times\mathbf{H}^*\right).
\end{aligned} \tag{S12}$$

Combining all above terms, we get the expression of the optical force as

$$\begin{aligned}
\mathbf{F}&=\frac{1}{4}\mathrm{Re}\left(\alpha_{ee}\right)\nabla\left|\mathbf{E}\right|^2+\omega\mu\mu_0\,\mathrm{Im}\left(\alpha_{ee}\right)\mathbf{P}+\frac{\omega\mu}{\varepsilon_0}\mathrm{Im}\left(\alpha_{ee}\right)\nabla\times\mathbf{L}_e \\
&+\frac{1}{4}\mathrm{Re}\left(\alpha_{mm}\right)\nabla\left|\mathbf{H}\right|^2+\omega\varepsilon\varepsilon_0\,\mathrm{Im}\left(\alpha_{mm}\right)\mathbf{P}+\frac{\omega\varepsilon}{\mu_0}\mathrm{Im}\left(\alpha_{mm}\right)\nabla\times\mathbf{L}_m \\
&-\frac{ck^4}{6\pi\sqrt{\varepsilon\mu}}\mathrm{Re}\left(\alpha_{ee}\alpha_{mm}{}^*\right)\mathbf{P}+\frac{ck^4}{12\pi\sqrt{\varepsilon\mu}}\mathrm{Im}\left(\alpha_{ee}\alpha_{mm}{}^*\right)\mathrm{Im}\left(\mathbf{E}\times\mathbf{H}^*\right) \\
&=-\nabla U+\sigma\mathbf{P}+\sigma_e\nabla\times\mathbf{L}_e+\sigma_m\nabla\times\mathbf{L}_m+\frac{\omega k^3}{12\pi}\mathrm{Im}\left(\alpha_{ee}\alpha_{mm}{}^*\right)\mathrm{Im}\left(\mathbf{E}\times\mathbf{H}^*\right),
\end{aligned} \tag{S13}$$

where $\nabla U=-\frac{1}{4}\mathrm{Re}(\alpha_{ee})\nabla|\mathbf{E}|^2-\frac{1}{4}\mathrm{Re}(\alpha_{mm})\nabla|\mathbf{H}|^2$, $\sigma_e=\omega\mu\mu_0c^2\mathrm{Im}\left(\alpha_{ee}\right)$, $\sigma_m=\omega\varepsilon\varepsilon_0c^2\mathrm{Im}\left(\alpha_{mm}\right)$, $\sigma=\frac{\sigma_e}{c^2}+\frac{\sigma_m}{c^2}-\frac{\omega k^3}{6\pi}\mathrm{Re}\left(\alpha_{ee}\alpha_{mm}^*\right)$.

Substituting the background field into the first term on the right side of Eq. (S13), i.e., the optical gradient force, we can get

$$
\begin{aligned}
-\nabla U &= \frac{1}{4}\mathrm{Re}\left(\alpha_{ee}\right)\nabla\left|\mathbf{E}\right|^2 + \frac{1}{4}\mathrm{Re}\left(\alpha_{mm}\right)\nabla\left|\mathbf{H}\right|^2 \\
&= \frac{1}{4}\mathrm{Re}\left(\alpha_{ee}\right)\nabla\left\{ A_E{}^2 \left\{1+\frac{K_E{}^2}{k^2\rho^2}\cos^2\theta\right\}\exp\left[-2\left(\frac{\rho-r_s}{w}\right)^2\right]\right\} \\
&+\frac{1}{4}\mathrm{Re}\left(\alpha_{mm}\right)\nabla\left\{\frac{A_E{}^2}{\omega^2\mu^2\mu_0^2\rho^2}\left\{\left[k\rho+2\frac{\rho-r_s}{kw^2}K_E+\frac{1}{k\rho}\left(m+\frac{2\rho^2}{w^2}\right)\right]^2\cos^2\theta\right.\right. \\
&\left.\left.+\left\{\left[k\rho+\frac{1}{k\rho}\left(m-1\right)K_E\right]^2+K_E{}^2\right\}\sin^2\theta\right\}\exp\left[-2\left(\frac{\rho-r_s}{w}\right)^2\right]\right\}.
\end{aligned}
\quad \text{(S14)}
$$

The optical force from the second term on the right side of Eq. (S13), which is associated with the real part of the Poynting momentum, can be determined as

$$
\begin{aligned}
\sigma\mathbf{P} &= \frac{\sigma}{2}\mathrm{Re}(\mathbf{E}\times\mathbf{H}^*) \\
&= \frac{\sigma}{2}\frac{A_E{}^2}{\omega\mu\mu_0\rho}\left\{\left[k\rho+2\frac{\rho-r_s}{kw^2}K_E+\frac{1}{k\rho}\left(m+\frac{2\rho^2}{w^2}\right)\right]\cos^2\theta\right. \\
&\left.+\left[k\rho+\frac{1}{k\rho}\left(m-1\right)K_E\right]\sin^2\theta\right\}\exp\left[-2\left(\frac{\rho-r_s}{w}\right)^2\right]\hat{\mathbf{e}}_z.
\end{aligned}
\quad \text{(S15)}
$$

Optical forces from third and fourth terms on the right side of Eq. (S13) arise from electric and magnetic parts of spin momenta, respectively, which can be expressed as

$$
\begin{aligned}
\sigma_e\left(\nabla\times\mathbf{L}_e\right) &= \sigma_e\frac{\varepsilon_0}{4\omega\mu i}\nabla\times\left(\mathbf{E}\times\mathbf{E}^*\right) \\
&= \sigma_e\frac{\varepsilon_0}{4\omega\mu i}\nabla\times\left\{\frac{iA_E{}^2K_E}{k\rho}\left[\sin\left(2\theta\right)\hat{\mathbf{e}}_\rho-2\cos^2\theta\hat{\mathbf{e}}_\varphi\right]\exp\left[-2\left(\frac{\rho-r_s}{w}\right)^2\right]\right\},
\end{aligned}
\quad \text{(S16)}
$$

$$
\begin{aligned}
\sigma_m\left(\nabla\times\mathbf{L}_m\right) &= \sigma_m\frac{\mu_0}{4\omega\varepsilon i}\nabla\times\left(\mathbf{H}\times\mathbf{H}^*\right) \\
&= \sigma_m\frac{\mu_0}{4\omega\varepsilon i}\nabla\times\left\{\frac{A_E{}^2}{\omega^2\mu^2\mu_0^2\rho^2}\left\{-iK_E\left[k\rho+2\frac{\rho-r_s}{kw^2}K_E+\frac{1}{k\rho}\left(m+\frac{2\rho^2}{w^2}\right)\right]\sin\left(2\theta\right)\hat{\mathbf{e}}_\rho\right.\right. \\
&\left.\left.-2iK_E\left[k\rho+\frac{1}{k\rho}\left(m-1\right)K_E\right]\sin^2\theta\hat{\mathbf{e}}_\varphi\right\}\exp\left[-2\left(\frac{\rho-r_s}{w}\right)^2\right]\right\}.
\end{aligned}
\quad \text{(S17)}
$$

The last term on the right side of Eq. (S13) is the optical force from the IPM $\mathrm{Im}\left(\mathbf{\Pi}\right)=\frac{1}{2c^2}\mathrm{Im}\left(\mathbf{E}\times\mathbf{H}^*\right)$. It is proportional to the time-averaged imaginary Poynting vector $\frac{1}{2}\mathrm{Im}\left(\mathbf{E}\times\mathbf{H}^*\right)$, and can be expressed as

$$\frac{\omega k^3}{12\pi}\mathrm{Im}\left(\alpha_{ee}\alpha_{mm}^*\right)\mathrm{Im}\left(\mathbf{E}\times\mathbf{H}^*\right)$$
$$=\frac{{A_E}^2k^3}{12\pi\mu\mu_0\rho}\mathrm{Im}\left(\alpha_{ee}\alpha_{mm}^*\right)\left\{\left\{-K_E\sin^2\theta+K_E\left[1+2\frac{\rho-r_s}{k^2w^2\rho}K_E+\frac{1}{k^2\rho^2}\left(m+\frac{2\rho^2}{w^2}\right)\right]\cos^2\theta\right\}\hat{\mathbf{e}}_\rho\right. \tag{S18}$$
$$\left.+\left\{\frac{K_E}{2}\left[2+\frac{1}{k^2\rho^2}(m-1)K_E\right]\sin(2\theta)\right\}\hat{\mathbf{e}}_\varphi\right\}\exp\left[-2\left(\frac{\rho-r_s}{w}\right)^2\right].$$

Each term in Eq. (S13) along the azimuthal direction can be expressed as

$$-\nabla U\big|_{\rho=r_s}^{\varphi}=\frac{1}{4}\frac{{A_E}^2m^2}{k^2{r_s}^3}(m-1)\sin(2\theta)\left\{-\mathrm{Re}(\alpha_{ee})+\frac{k^2}{\omega^2\mu^2\mu_0^2}\mathrm{Re}(\alpha_{mm})\left[3-\frac{4}{m}-\frac{4{r_s}^2}{m^2w^2}\right.\right.$$
$$\left.\left.-\frac{1}{k^2{r_s}^2}\left(\frac{4{r_s}^4}{m^2w^4}+\frac{4{r_s}^2}{mw^2}-m^2+2m\right)\right]\right\}, \tag{S19}$$

$$-\sigma\mathbf{P}\big|_{\rho=r_s}^{\varphi}=0, \tag{S20}$$

$$\sigma_e\left(\nabla\times\mathbf{L}_e\right)\big|_{\rho=r_s}^{\varphi}=0, \tag{S21}$$

$$\sigma_m\left(\nabla\times\mathbf{L}_m\right)\big|_{\rho=r_s}^{\varphi}=0, \tag{S22}$$

$$\frac{\omega k^3}{12\pi}\mathrm{Im}\left(\alpha_{ee}\alpha_{mm}^*\right)\mathrm{Im}\left(\mathbf{E}\times\mathbf{H}^*\right)\Big|_{\rho=r_s}^{\varphi}$$
$$=\frac{{A_E}^2k^3}{24\pi\mu\mu_0\rho}\mathrm{Im}\left(\alpha_{ee}\alpha_{mm}^*\right)K_E\left[2+\frac{1}{k^2\rho^2}(m-1)K_E\right]\sin(2\theta)\exp\left[-2\left(\frac{\rho-r_s}{w}\right)^2\right]\Bigg|_{\rho=r_s}^{\varphi} \tag{S23}$$
$$=\frac{{A_E}^2k^3m}{24\pi\mu\mu_0r_s}\mathrm{Im}\left(\alpha_{ee}\alpha_{mm}^*\right)\left(2+\frac{m^2-m}{k^2{r_s}^2}\right)\sin\left[2\theta_0+2(m-1)\varphi\right].$$

In this work, we compare optical forces from the dipole theory with that obtained from the Minkowski stress tensor using the electromagnetic field from simulations of a particle-free model. The optical gradient force [Eq. (S19)] is zero at $m = 1$. It is much smaller than the polarization-topology optical force (PTOF) from the IPM [Eq. (S23)] when $m > 1$, as shown in Fig. S6.

In the case of the dipole approximation, electric and magnetic polarizabilities can be computed using Mie coefficients as

$$a_v=\frac{{m_p}^2j_v(m_px_p)\left[x_pj_v(x_p)\right]'-j_v(x_p)\left[m_px_pj_v(m_px_p)\right]'}{{m_p}^2j_v(m_px_p)\left[x_ph_v^{(1)}(x_p)\right]'-h_v^{(1)}(x_p)\left[m_px_pj_v(m_px_p)\right]'}, \tag{S24}$$

$$b_v = \frac{j_v\left(m_p x_p\right)\left[x_p j_v\left(x_p\right)\right]' - j_v\left(x_p\right)\left[m_p x_p j_v\left(m_p x_p\right)\right]'}{j_v\left(m_p x_p\right)\left[x_p h_v^{(1)}\left(x_p\right)\right]' - h_v^{(1)}\left(x_p\right)\left[m_p x_p j_v\left(m_p x_p\right)\right]'}, \tag{S25}$$

where $m_p = n_p / n$ is the relative refractive index; $x_p = kr$ is the normalized particle radius, with r being the particle radius. Additionally, $j_v(\cdot)$ and $h_v^{(1)}(\cdot)$ denote the first-order spherical Bessel functions and Hankel functions, respectively. The electric and magnetic polarizabilities can be expressed as

$$\alpha_{ee} = i\frac{6\pi\varepsilon\varepsilon_0}{k^3}a_1, \tag{S26}$$

$$\alpha_{mm} = i\frac{6\pi\mu\mu_0}{k^3}b_1. \tag{S27}$$

Supplementary Note 3: Derivation of the optical force using the multipole theory

Considering the physical process, in any monochromatic optical field, the time-averaged optical force exerted on a particle can be decomposed into two distinct components, the interception (extinction) force $\langle \mathbf{F}_{\text{int}} \rangle$ and recoil force $\langle \mathbf{F}_{\text{rec}} \rangle$. Based on the T-matrix method and the multipole field theory, the optical force can be explicitly expressed in terms of the induced electric and magnetic multipole moments of different orders. To be specific, the interception force $\langle \mathbf{F}_{\text{int}} \rangle$ is given by

$$\langle \mathbf{F}_{\text{int}} \rangle = \sum_{l=1}^{\infty}\left[\langle \mathbf{F}_{\text{int}}^{e(l)} \rangle + \langle \mathbf{F}_{\text{int}}^{m(l)} \rangle\right], \tag{S28}$$

and the recoil force $\langle \mathbf{F}_{\text{rec}} \rangle$ reads

$$\langle \mathbf{F}_{\text{rec}} \rangle = \sum_{l=1}^{\infty}\left[\langle \mathbf{F}_{\text{rec}}^{e(l)} \rangle + \langle \mathbf{F}_{\text{rec}}^{m(l)} \rangle + \langle \mathbf{F}_{\text{rec}}^{x(l)} \rangle\right], \tag{S29}$$

where the superscripts "*e*", "*m*", and "*x*" denote contributions arising from electric multipoles, magnetic multipoles and hybrid term, respectively. The electric and magnetic multipoles of order l are described by totally symmetric and traceless rank-l tensors $\overleftrightarrow{\mathbb{O}}_{\text{elec}}^{(l)}$ and $\overleftrightarrow{\mathbb{O}}_{\text{mag}}^{(l)}$, respectively. The lower-order cases with l = 1, 2, 3, and 4 correspond to the dipole, quadrupole, octupole, and hexadecapole, respectively. The multiple tensor contraction between two tensors of ranks l and l',

denoted by $\overset{(m)}{\cdot\cdot}$, yields a tensor of rank $l + l' - 2m$ defined as

$$\overleftrightarrow{\mathbb{A}}^{(l)} \overset{(m)}{\cdot\cdot} \overleftrightarrow{\mathbb{B}}^{(l')} = \mathbb{A}^{(l)}_{i_1 i_2 \cdots i_{l-m} k_1 k_2 \cdots k_{m-1} k_m} \mathbb{B}^{(l')}_{k_m k_{m-1} \cdots k_2 k_1 j_{m+1} \cdots j_{l'-1} j_{l'}} ,\ 0 \le m \le \min\left[l, l'\right], \tag{S30}$$

with the summation over repeated indices implied (Einstein summation convention). Here the tensor contraction is made consecutively over two nearest indices in two index sequences. Eqs. (S28) and (S29) constitute the complete multipole expansion of optical force on any particle, up to arbitrary orders of multipoles.

The totally symmetric and traceless multipole moments, $\overleftrightarrow{\mathbb{O}}^{(l)}_{\text{elec}}$ and $\overleftrightarrow{\mathbb{O}}^{(l)}_{\text{mag}}$, which are therefore referred to as 2^l-pole moments, are derived based on the theory of multipole fields

$$\overleftrightarrow{\mathbb{O}}^{(l)}_{\text{elec (mag)}} = \gamma^{(l)}_{\text{elec (mag)}} \sum_{m=0}^{\lfloor (l-1)/2 \rfloor} d_{l,m} k^{2m} \overleftrightarrow{\mathbb{N}}^{(l,m)}_{\text{elec (mag)}} , \tag{S31}$$

where $d_{l,m} = \frac{1}{4^m} \frac{l!}{m!} \frac{\Gamma\left(l - m + \frac{1}{2}\right)}{\Gamma\left(l + \frac{1}{2}\right)\Gamma(l-2m)} \frac{1}{l}$, with $d_{l,0} = 1$, $\lfloor x \rfloor$ giving the greatest integer less than or equaling x, and $\Gamma(x)$ denoting the Gamma function. The lower-order cases with l = 1, 2, 3, and 4, correspond to the dipole, quadrupole, octupole, and hexadecapole moments, respectively. For instance, $\overleftrightarrow{\mathbb{O}}^{(1)}_{\text{mag}}$ reduces to the electric dipole moment $\overleftrightarrow{p}$, $\overleftrightarrow{\mathbb{O}}^{(1)}_{\text{mag}}$ is the magnetic dipole moment $\overleftrightarrow{m}$, $\overleftrightarrow{\mathbb{O}}^{(2)}_{\text{mag}}$ represents the electric quadrupole moment $\overleftrightarrow{Q}^{(e)}$, and $\overleftrightarrow{\mathbb{O}}^{(2)}_{\text{mag}}$ delineates the magnetic quadrupole moment $\overleftrightarrow{Q}^{(m)}$. The electric and magnetic polarizabilities, $\gamma^{(l)}_{\text{elec}}$ and $\gamma^{(l)}_{\text{mag}}$, depend on the Mie coefficients a_l and b_l of a spherical particle through

$$\gamma^{(l)}_{\text{elec}} = \frac{4l(2l+1)!}{2^l (l+1)!} \frac{i\pi\varepsilon\varepsilon_0 a_l}{k^{2l+1}} , \tag{S32}$$

$$\gamma^{(l)}_{\text{mag}} = \frac{4l(2l+1)!}{2^l (l+1)!} \frac{i\pi b_l}{\mu\mu_0 k^{2l+1}} , \tag{S33}$$

with

$$c_{l,m} = \frac{(-1)^m}{4^m} \frac{l!}{m!} \frac{\Gamma\left(l - m + \frac{1}{2}\right)}{\Gamma\left(l + \frac{1}{2}\right)\Gamma(l-2m)} \frac{(l-2m)}{l^2} = \frac{(-1)^m (l-2m)}{l} d_{l,m} , \tag{S34}$$

$$f_{l,m}=\frac{(-1)^m}{4^m}\frac{l!}{m!}\frac{\Gamma\left(l-m+\frac{1}{2}\right)}{\Gamma\left(l+\frac{1}{2}\right)\Gamma(l-2m)}\frac{(l-2m+1)(2l-2m+1)}{l(l+1)(2l+1)}, \tag{S35}$$

$$g_{l,m}=\frac{(-1)^m}{4^m}\frac{l!}{m!}\frac{\Gamma\left(l-m+\frac{1}{2}\right)}{\Gamma\left(l+\frac{1}{2}\right)\Gamma(l-2m)}\frac{(l-2m)(l-2m-1)}{l(l+1)(2l+1)}, \tag{S36}$$

$$h_{l,m}=\frac{(-1)^m}{4^m}\frac{l!}{m!}\frac{\Gamma\left(l-m+\frac{1}{2}\right)}{\Gamma\left(l+\frac{1}{2}\right)\Gamma(l-2m)}\frac{(l-2m)^2}{l^3}. \tag{S37}$$

After algebraic computation, the interception parts $\left\langle \mathbf{F}_{\text{int}}^{e(l)} \right\rangle$ and $\left\langle \mathbf{F}_{\text{int}}^{m(l)} \right\rangle$ of the optical force involving order l multipoles can be rewritten as

$$\left\langle \mathbf{F}_{\text{int}}^{e(l)} \right\rangle=\frac{1}{2l!}\sum_{m=0}^{\lfloor (l-1)/2 \rfloor} c_{l,m}k^{4m}\,\text{Re}\left[\gamma_{\text{elec}}^{(l)}\mathbf{t}_{\text{elec}}^{(l-2m)}\right], \tag{S38}$$

$$\left\langle \mathbf{F}_{\text{int}}^{m(l)} \right\rangle=\frac{1}{2l!}\sum_{m=0}^{\lfloor (l-1)/2 \rfloor} c_{l,m}k^{4m}\,\text{Re}\left[\gamma_{\text{mag}}^{(l)}\mathbf{t}_{\text{mag}}^{(l-2m)}\right], \tag{S39}$$

where

$$\begin{aligned}\mathbf{t}_{\text{elec}}^{(n)} &\equiv \left[\nabla^{(n)}\mathbf{E}^*\right] \overset{(n)}{\cdot\cdot}\, \overleftrightarrow{\mathbb{M}}_{\text{elec}}^{(n)} \\ &=\frac{1}{2}\left[\nabla D_{\text{ee}}^{(n)}-\nabla\times\mathbf{S}_{\text{ee}}^{(n)}-2i\omega\,\text{Re}\,\mathbf{S}_{\text{em}}^{(n)}\right] \\ &-\frac{(n-1)\omega^2}{2n}\left[\nabla D_{\text{mm}}^{(n-1)}-\nabla\times\mathbf{S}_{\text{mm}}^{(n-1)}-\frac{2i\omega}{c^2}\text{Re}\,\mathbf{S}_{\text{em}}^{(n-1)}\right],\end{aligned} \tag{S40}$$

$$\begin{aligned}\mathbf{t}_{\text{mag}}^{(n)} &\equiv \left[\nabla^{(n)}\mathbf{B}^*\right] \overset{(n)}{\cdot\cdot}\, \overleftrightarrow{\mathbb{M}}_{\text{mag}}^{(n)} \\ &=\frac{1}{2}\left[\nabla D_{\text{mm}}^{(n)}-\nabla\times\mathbf{S}_{\text{mm}}^{(n)}-\frac{2i\omega}{c^2}\text{Re}\,\mathbf{S}_{\text{em}}^{(n)}\right] \\ &-\frac{(n-1)\omega^2}{2nc^4}\left[\nabla D_{\text{ee}}^{(n-1)}-\nabla\times\mathbf{S}_{\text{ee}}^{(n-1)}-2i\omega\,\text{Re}\,\mathbf{S}_{\text{em}}^{(n-1)}\right].\end{aligned} \tag{S41}$$

The field moments in the reciprocal space are defined as follows.

$$D_{\text{ee}}^{(n)}=\left(\nabla^{(n-1)}\mathbf{E}\right)\overset{(n)}{:}\left(\nabla^{(n-1)}\mathbf{E}^*\right), \tag{S42}$$

$$D_{\mathrm{mm}}^{(n)}=\left(\nabla^{(n-1)}\mathbf{B}\right)\overset{(n)}{:}\left(\nabla^{(n-1)}\mathbf{B}^{*}\right), \tag{S43}$$

$$\mathbf{S}_{\mathrm{ee}}^{(n)}=\left[\left(\nabla^{(n-1)}\mathbf{E}\right)\overset{(n-1)}{:}\left(\nabla^{(n-1)}\mathbf{E}^{*}\right)\right]\overset{(2)}{:}\vec{\boldsymbol{\epsilon}}\,, \tag{S44}$$

$$\mathbf{S}_{\mathrm{mm}}^{(n)}=\left[\left(\nabla^{(n-1)}\mathbf{B}\right)\overset{(n-1)}{:}\left(\nabla^{(n-1)}\mathbf{B}^{*}\right)\right]\overset{(2)}{:}\vec{\boldsymbol{\epsilon}}\,, \tag{S45}$$

$$\mathbf{S}_{\mathrm{em}}^{(n)}=\left[\left(\nabla^{(n-1)}\mathbf{E}\right)\overset{(n-1)}{:}\left(\nabla^{(n-1)}\mathbf{B}^{*}\right)\right]\overset{(2)}{:}\vec{\boldsymbol{\epsilon}}\,, \tag{S46}$$

where $\vec{\boldsymbol{\epsilon}}$ is the Levi-Civita tensor, whose components ϵ_{ijk} are antisymmetric with respect to the permutation of any pair of indices. The second kind of multiple tensor contraction $\overset{(m)}{:}$ is denoted by

$$\overset{\leftrightarrow}{\mathbb{A}}^{(n)}\overset{(m)}{:}\overset{\leftrightarrow}{\mathbb{B}}^{(n')}=\mathbb{A}_{k_1k_2\cdots k_m i_{m+1}i_{m+2}\cdots i_n}^{(n)}\mathbb{B}_{k_1k_2\cdots k_m j_{m+1}j_{m+2}\cdots j_{n'}}^{(n')}\,,\; 0\le m\le\min\left[n,n'\right]. \tag{S47}$$

The electric and magnetic parts of the recoil force can be worked out to give

$$\begin{aligned}\left\langle\mathbf{F}_{\mathrm{rec}}^{e(l)}\right\rangle=-\frac{c_l k^{2l+3}}{4\pi\varepsilon\varepsilon_0}&\left\{\sum_{m=0}^{\lfloor(l-1)/2\rfloor}f_{l,m}k^{4m}\,\mathrm{Im}\left[\eta_{\mathrm{elec}}^{(l)}\mathbf{t}_{\mathrm{ee}}^{(l-2m)*}\right]\right.\\&\left.+\sum_{m=0}^{\lfloor(l-2)/2\rfloor}g_{l,m}k^{4m+2}\,\mathrm{Im}\left[\eta_{\mathrm{elec}}^{(l)}\mathbf{t}_{\mathrm{ee}}^{(l-2m-1)}\right]\right\},\end{aligned} \tag{S48}$$

$$\begin{aligned}\left\langle\mathbf{F}_{\mathrm{rec}}^{m(l)}\right\rangle=-\frac{c_l k^{2l+3}}{4\pi\varepsilon\varepsilon_0 c^2}&\left\{\sum_{m=0}^{\lfloor(l-1)/2\rfloor}f_{l,m}k^{4m}\,\mathrm{Im}\left[\eta_{\mathrm{mag}}^{(l)}\mathbf{t}_{\mathrm{mm}}^{(l-2m)*}\right]\right.\\&\left.+\sum_{m=0}^{\lfloor(l-2)/2\rfloor}g_{l,m}k^{4m+2}\,\mathrm{Im}\left[\eta_{\mathrm{mag}}^{(l)}\mathbf{t}_{\mathrm{mm}}^{(l-2m-1)}\right]\right\},\end{aligned} \tag{S49}$$

where

$$\begin{aligned}\mathbf{t}_{\mathrm{ee}}^{(n)}&\equiv\overleftrightarrow{\mathbb{M}}_{\mathrm{elec}}^{(n)}\overset{(n)}{\cdot\cdot}\overleftrightarrow{\mathbb{M}}_{\mathrm{elec}}^{(n+1)*}=\overleftrightarrow{\mathbb{M}}_{\mathrm{elec}}^{(n+1)*}\overset{(n)}{\cdot\cdot}\overleftrightarrow{\mathbb{M}}_{\mathrm{elec}}^{(n)}\\&=\frac{1}{2}\left[\nabla D_{\mathrm{ee}}^{(n)}-\nabla\times\mathbf{S}_{\mathrm{ee}}^{(n)}-2i\omega\,\mathrm{Re}\,\mathbf{S}_{\mathrm{em}}^{(n)}\right]+\frac{i\omega}{(n+1)}\mathbf{S}_{\mathrm{em}}^{(n)}\\&-\frac{(n-1)\omega^2}{2(n+1)}\left[\nabla D_{\mathrm{mm}}^{(n-1)}-\nabla\times\mathbf{S}_{\mathrm{mm}}^{(n-1)}-\frac{2i\omega}{c^2}\mathrm{Re}\,\mathbf{S}_{\mathrm{em}}^{(n-1)}\right],\end{aligned} \tag{S50}$$

$$\mathbf{t}_{\rm mm}^{(n)} \equiv \overleftrightarrow{\mathbb{M}}_{\rm mag}^{(n)} \overset{(n)}{\cdot\cdot} \overleftrightarrow{\mathbb{M}}_{\rm mag}^{(n+1)*} = \overleftrightarrow{\mathbb{M}}_{\rm mag}^{(n+1)*} \overset{(n)}{\cdot\cdot} \overleftrightarrow{\mathbb{M}}_{\rm mag}^{(n)}$$

$$= \frac{1}{2}\left[\nabla D_{\rm mm}^{(n)} - \nabla\times\mathbf{S}_{\rm mm}^{(n)} - \frac{2i\omega}{c^2}\mathrm{Re}\,\mathbf{S}_{\rm em}^{(n)}\right] + \frac{i\omega}{(n+1)c^2}\mathbf{S}_{\rm em}^{(n)*} \tag{S51}$$

$$-\frac{(n-1)\omega^2}{2(n+1)c^4}\left[\nabla D_{\rm ee}^{(n-1)} - \nabla\times\mathbf{S}_{\rm ee}^{(n-1)} - 2i\omega\,\mathrm{Re}\,\mathbf{S}_{\rm em}^{(n-1)}\right],$$

with $c_l = 2^{l+1}\left(l+2\right)/\left(2l+3\right)!$, $\eta_{\rm elec}^{(l)} = \gamma_{\rm elec}^{(l+1)}\gamma_{\rm elec}^{(l)*}$, and $\eta_{\rm mag}^{(l)} = \gamma_{\rm mag}^{(l+1)}\gamma_{\rm mag}^{(l)*}$.

The hybrid term of the recoil force can be cast into

$$\left\langle \vec{\mathbf{F}}_{\rm rec}^{x(l)} \right\rangle = \frac{1}{4\pi\varepsilon\varepsilon_0 c}\frac{2^l k^{2l+2}}{l(2l+1)!}\sum_{m=0}^{\lfloor (l-1)/2 \rfloor} h_{l,m}k^{4m}\,\mathrm{Re}\left[\eta_{\rm hyb}^{(l)}\mathbf{t}_{\rm em}^{(l-2m)}\right], \tag{S52}$$

where

$$\mathbf{t}_{\rm em}^{(n)} \equiv \left[\overleftrightarrow{\mathbb{M}}_{\rm elec}^{(n)} \overset{(n-1)}{\cdot\cdot} \overleftrightarrow{\mathbb{M}}_{\rm mag}^{(n)\,*}\right]^{(2)} \cdot\cdot\, \vec{\boldsymbol{\epsilon}}^{(n)}$$

$$= \frac{i(n-1)\omega}{nc^2}\left[c^2\mathbf{Z}_{\rm mm}^{(n-1)} - \mathbf{Z}_{\rm ee}^{(n-1)*}\right] + \frac{i(n-1)(n-2)\omega k^2}{n^2c^2}\left[c^2\mathbf{Z}_{\rm mm}^{(n-2)} - \mathbf{Z}_{\rm ee}^{(n-2)*}\right] \tag{S53}$$

$$-\mathbf{S}_{\rm em}^{(n)} - \frac{(n-1)k^2}{n^2}\mathbf{S}_{\rm em}^{(n-1)*} + \frac{(n-1)(n-2)k^4}{2n^2}\mathbf{S}_{\rm em}^{(n-2)},$$

with $\eta_{\rm hyb}^{(l)} = \gamma_{\rm elec}^{(l)}\gamma_{\rm mag}^{(l)*}$, and

$$\mathbf{Z}_{\rm ee}^{(n)} = \frac{1}{2}\left[\nabla D_{\rm ee}^{(n)} - \nabla\times\mathbf{S}_{\rm ee}^{(n)} - 2i\omega\,\mathrm{Re}\,\mathbf{S}_{\rm em}^{(n)}\right], \tag{S54}$$

$$\mathbf{Z}_{\rm mm}^{(n)} = \frac{1}{2}\left[\nabla D_{\rm mm}^{(n)} - \nabla\times\mathbf{S}_{\rm mm}^{(n)} - \frac{2i\omega}{c^2}\mathrm{Re}\,\mathbf{S}_{\rm em}^{(n)}\right], \tag{S55}$$

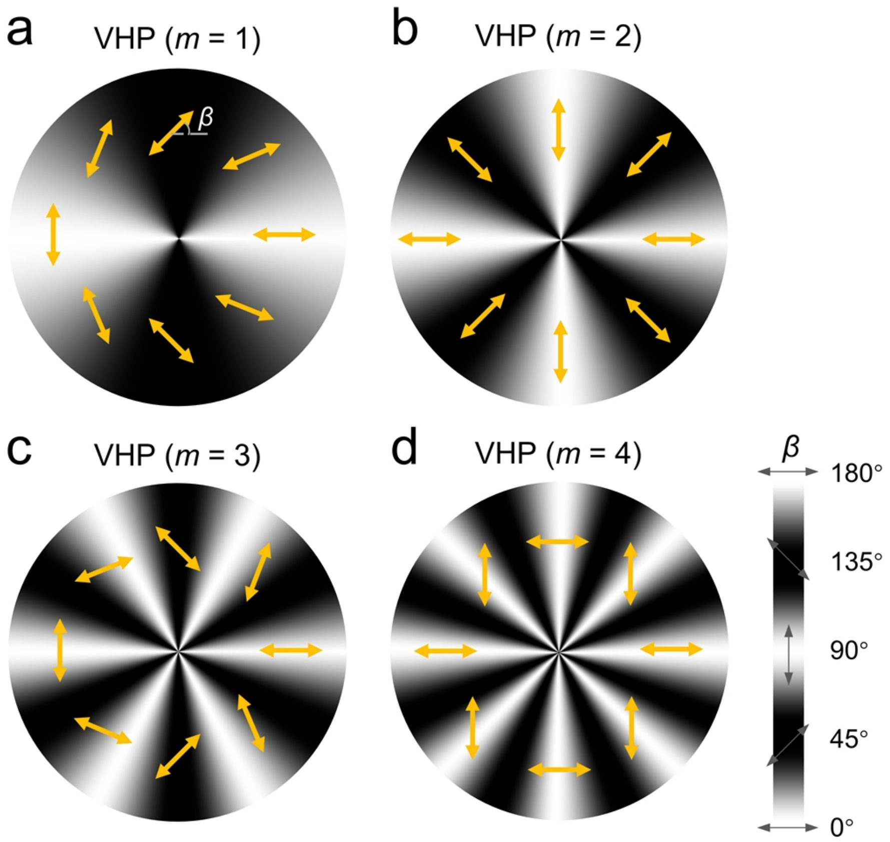

Figure S1. Characterization of the vector light field. (a−d) Illustration of vortex half-wave plates with different orders. Yellow arrows indicate fast-axis orientations of waveplates.

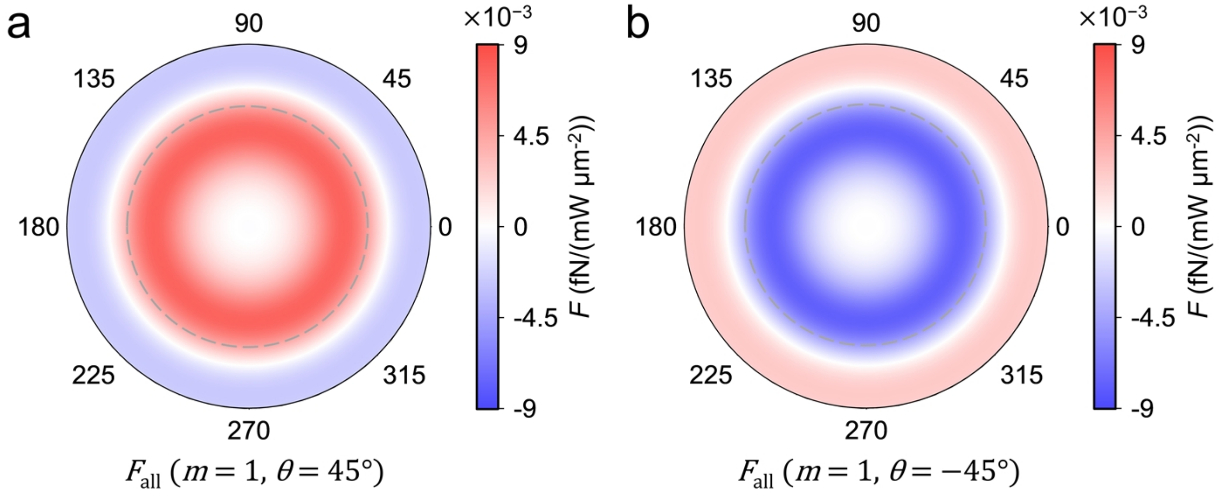

Figure S2. Simulation of total optical forces at *m* = 1. Simulated total optical force (F_{all}) on the 100-nm nanoparticle using the Minkowski stress tensor in the doughnut-shaped beam [simulation radius, 9 µm; radius of the circle with the maximum intensity (dashed line), 6 µm] when $m = 1$ and (a) $\theta = 45°$ or (b) $\theta = -45°$. The laser intensity in the circle is set to 1 mW/µm^2. Both F_{all} and F_{PTC} (Fig. 2) exhibit sign reversal for opposite θ. The different $\nabla|E|$ across the circle generates opposing optical forces on either side. Although the calculated forces for 100-nm particles are exceedingly small, the 3-µm particles employed in our experiments experience detectable forces on the order of 10 fN under a laser intensity of approximately 0.79 mW/µm^2.

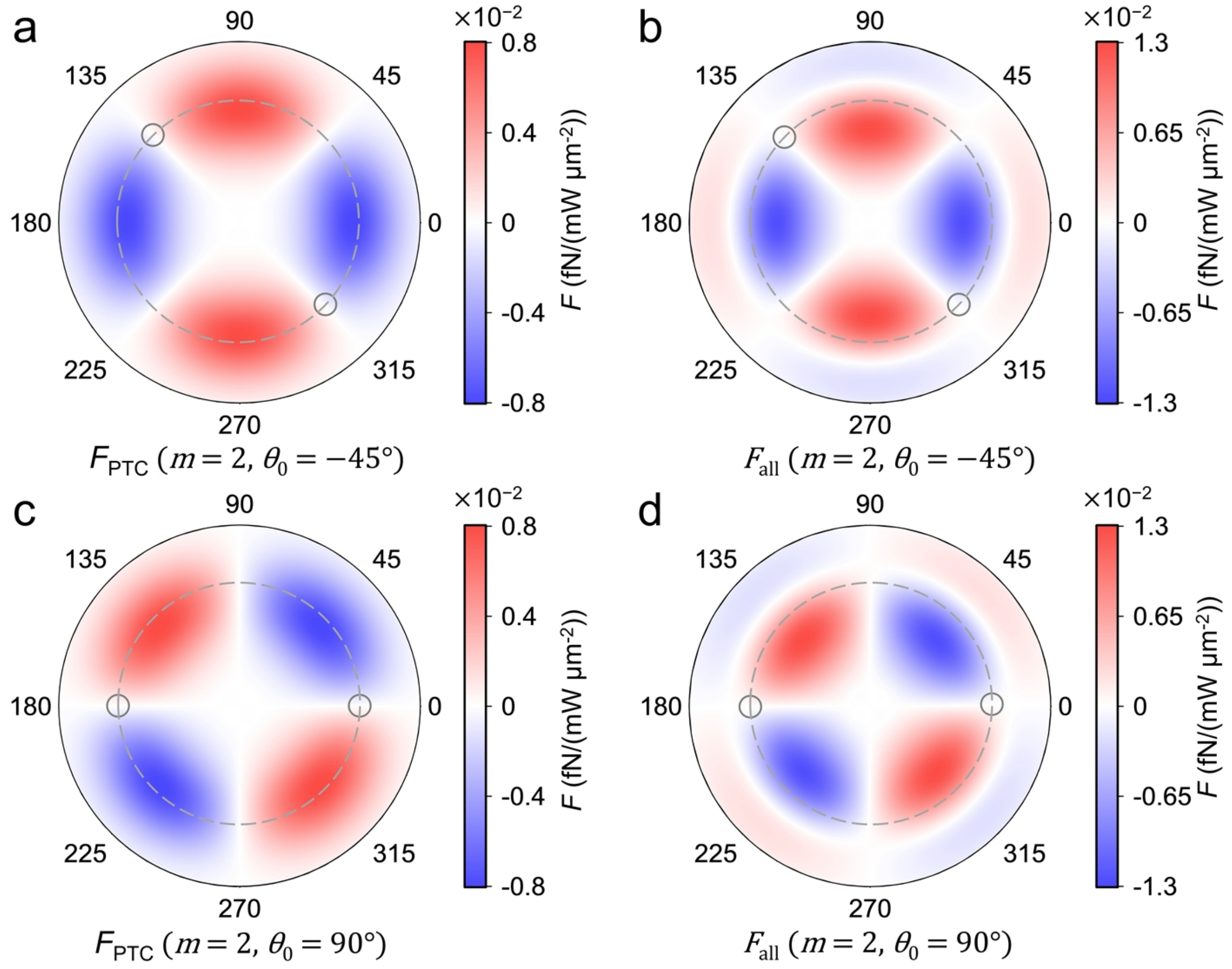

Figure S3. Simulation of optical forces from the imaginary Poynting momentum. Simulated (a) polarization-topology optical force (PTOF, F_{PTC}) and (b) F_{all} on the 100-nm nanoparticle using the Minkowski stress tensor in the doughnut-shaped beam when $m = 2$ and $\theta_{\varphi = 0°} = -45°$. Simulated (c) F_{PTC} and (d) F_{all} on the 100-nm nanoparticle when $m = 2$ and $\theta_{\varphi = 0°} = 90°$. The stable trapping position varies with $\theta_{\varphi = 0°}$, indicating a feasible way to rotate particle pairs (or arrays for $m > 2$). Since F_{all} comprises both F_{PTC} and $F_{\nabla|E|}$, with $F_{\nabla|E|}$ vanishing at the maximum intensity and exhibiting opposite directions on either side of the intensity peak, a reversal in F_{all} occurs when $F_{\nabla|E|}$ exceeds F_{PTC} in magnitude and opposes the direction of F_{all}. This explains the observed reversal in F_{all} in $x > 0$ regions.

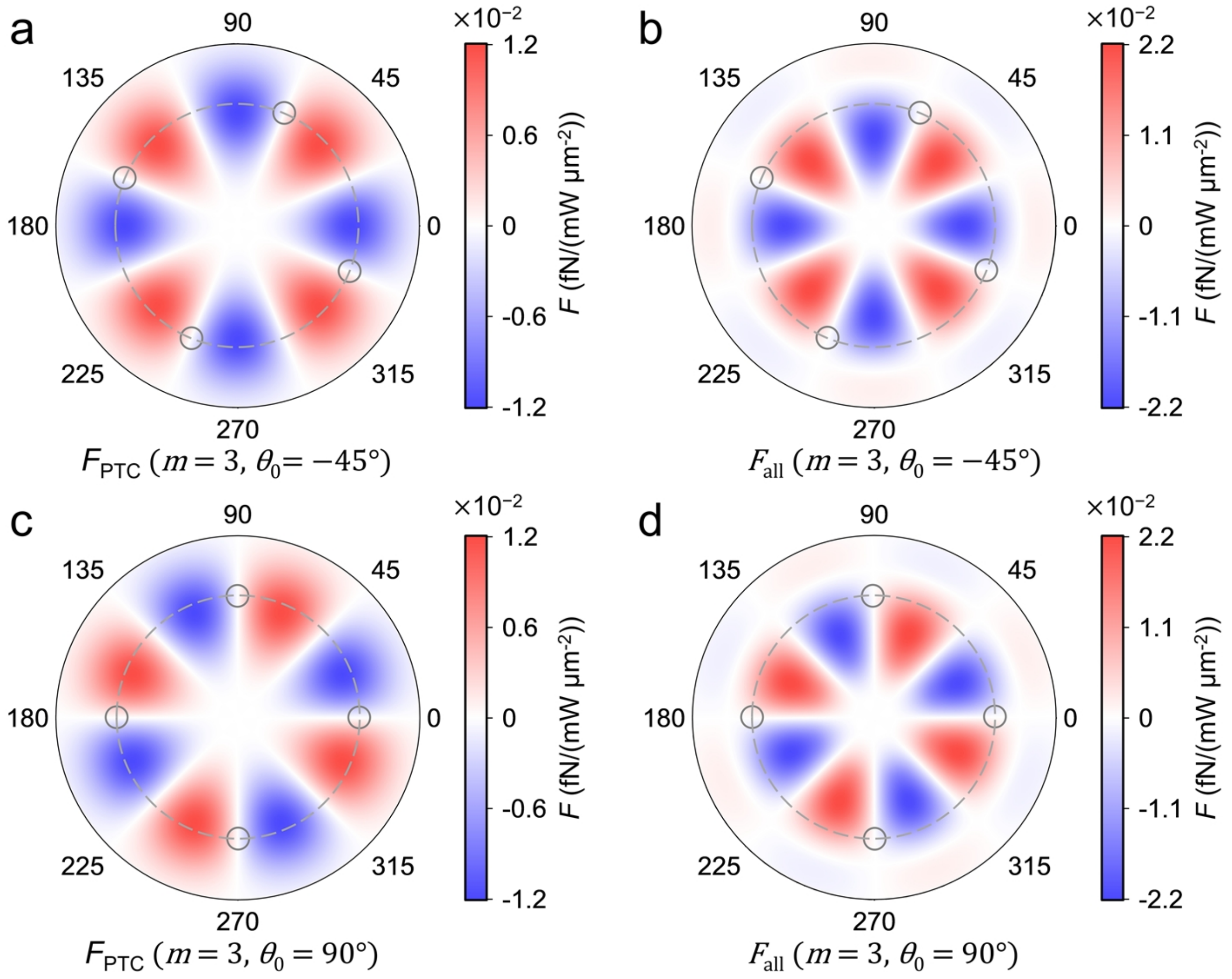

Figure S4. Simulation of optical forces from the imaginary Poynting momentum. Simulated (a) F_{PTC} and (b) F_{all} on the 100-nm nanoparticle using the Minkowski stress tensor in the doughnut-shaped beam when $m = 3$ and $\theta_0 = -45°$. Simulated (c) F_{PTC} and (d) F_{all} on the 100-nm nanoparticle when $m = 3$ and $\theta_0 = 90°$. The number of potential wells increases to four when m is changed from two (Fig. S3) to three.

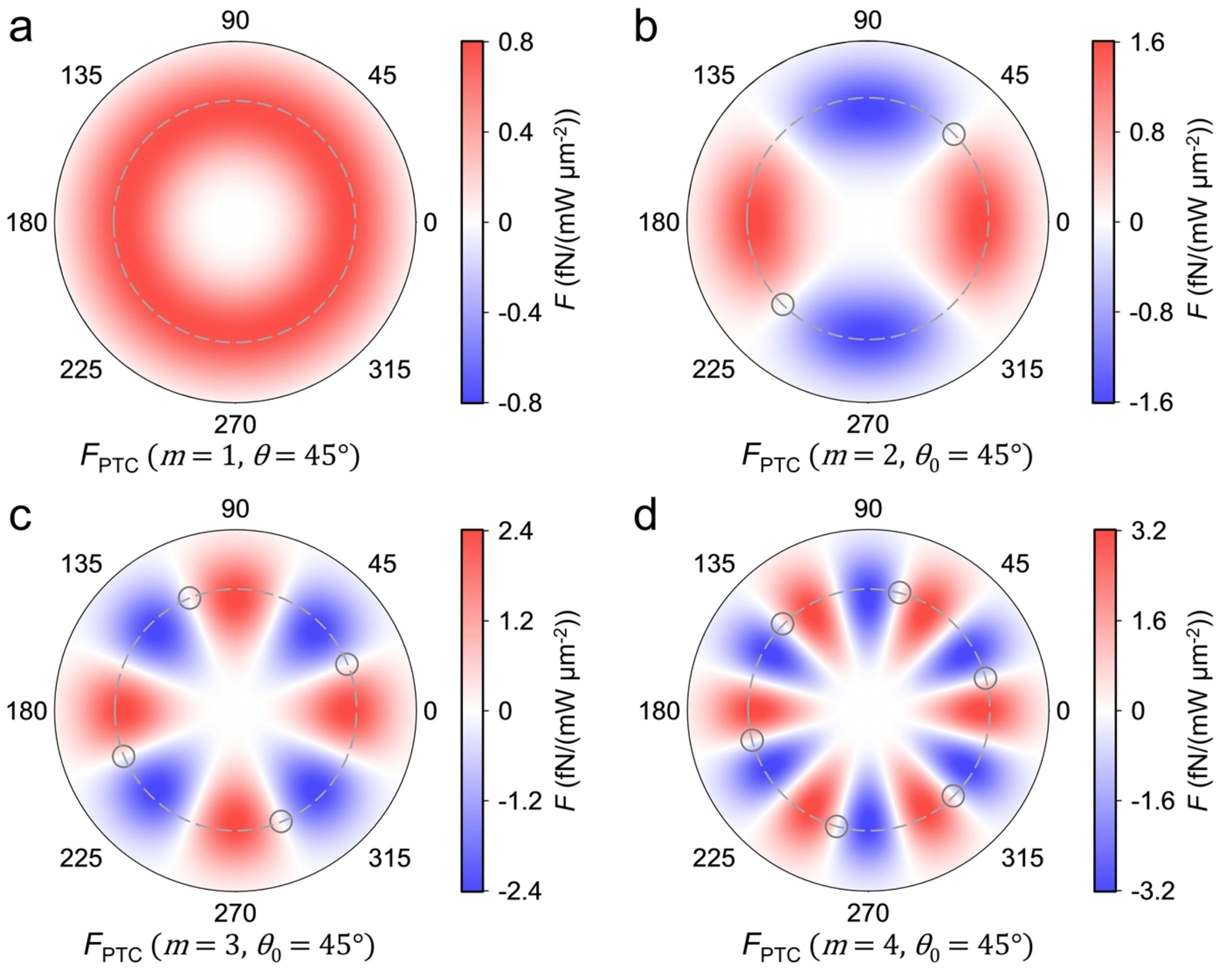

Figure S5. Simulation of optical forces on gold nanoparticles from the imaginary Poynting momentum. (a–d) Simulated F_{PTC} on the 100-nm nanoparticle using the Minkowski stress tensor in the doughnut-shaped beam when m = 1, 2, 3, 4, respectively. θ_0 = 45° for m = 1, 2, 3 and 4.

The phenomena for the gold nanoparticle are the same as the polystyrene one, as shown in Fig. S5. Particles rotate at $m = 1$, while they can be trapped under the balance of positive and negative PTOFs at $m > 1$. The number of potential wells increases linearly with the PTC, following the relationship $2(m - 1)$. It is worth noting that, under identical conditions, the PTOF acting on the gold nanoparticle is two orders of magnitude greater than that on the polystyrene particle, which arises from the larger imaginary part of the refractive index of gold.

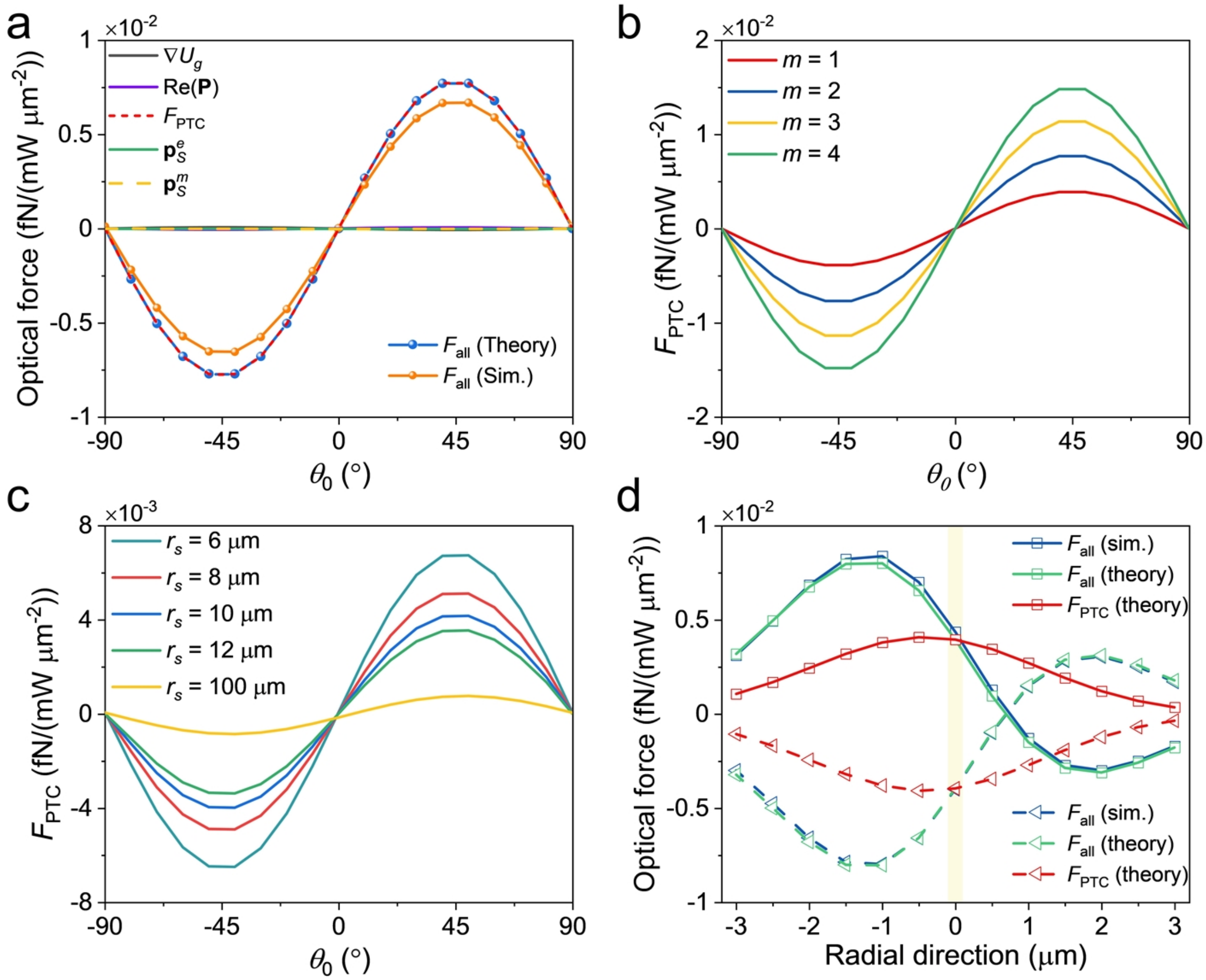

Figure S6. Simulation of optical forces from the imaginary Poynting momentum. (a) Different types of optical forces on the 100-nm nanoparticle under various polarization angles when $m = 2$. Forces from the intensity gradient (∇U_g), the real part of the Poynting momentum [Re(**P**)] and the Belinfante spin momentum ($\mathbf{p}_S^e$ and $\mathbf{p}_S^m$) vanish due to the $|E|$ symmetry. (b) F_{PTC} on the 100-nm nanoparticle under different polarization angles when $m = 1$, 2, 3 and 4. The PTOF is proportional to m. (c) F_{PTC} under different radii of the circle, r_s. The PTOF decreases as the circle radius increases, owing to the reduced rate of change of polarization. (d) Comparison of F_{PTC} and F_{all} when $m = 1$, $\theta = 45°$ (solid line) and $-45°$ (dashed line). The calculation is performed by normalizing the intensity of the circle to 1 mW/μm^2.

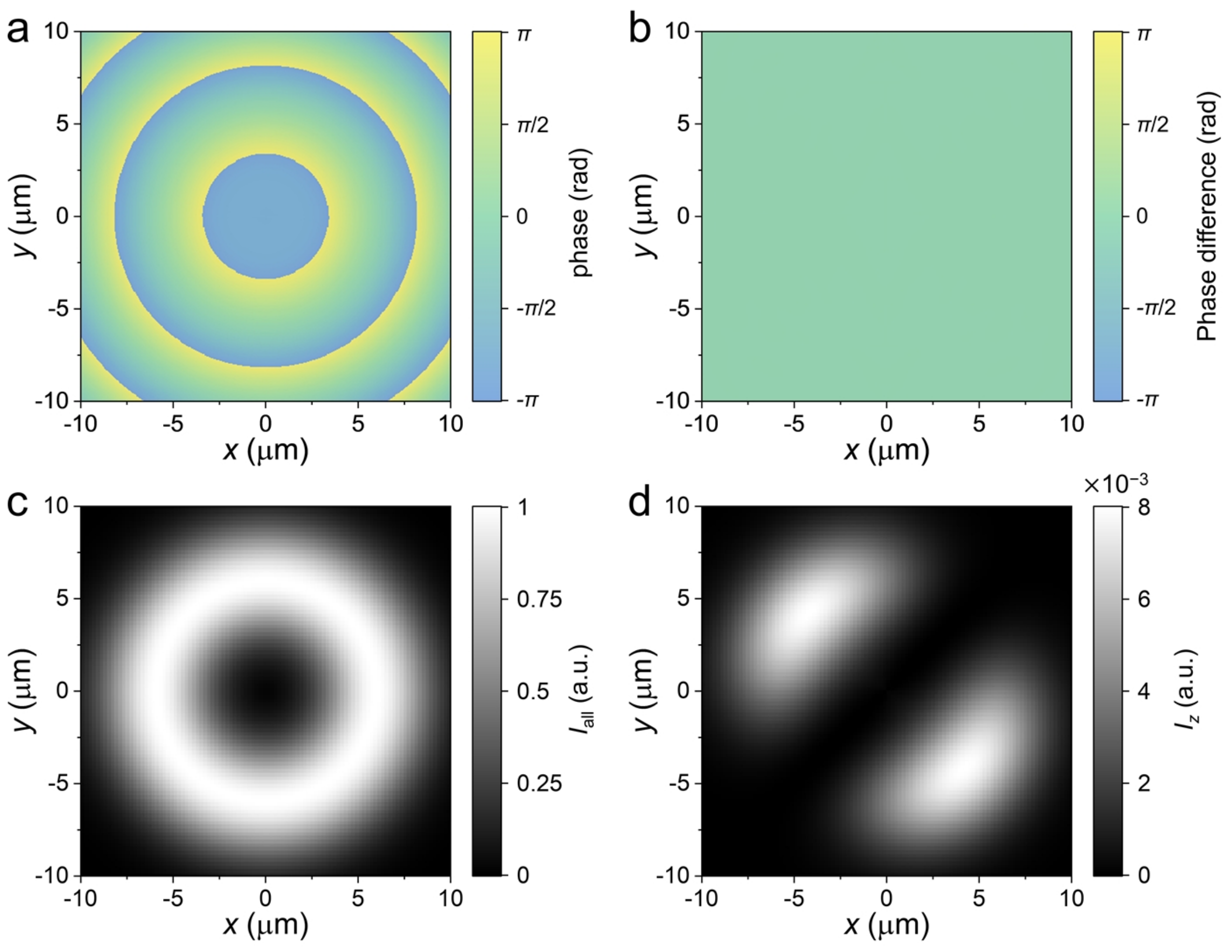

Figure S7. Properties of the vector beam after focusing. (a, b) Phase and phase difference between the components in the *x* and *y* directions. (c, d) Distributions of the total intensity and the components of intensity in the *z* direction of the focused beam. The vector beam (m = 2, θ_0 = 45°) is focused through an objective lens (NA = 0.25). The component of the intensity in the *z* direction is two orders of magnitude smaller than the total intensity. Properties of the vector beam are calculated using the Richards-Wolf equation. The observed plane is 50 μm from the focal plane.

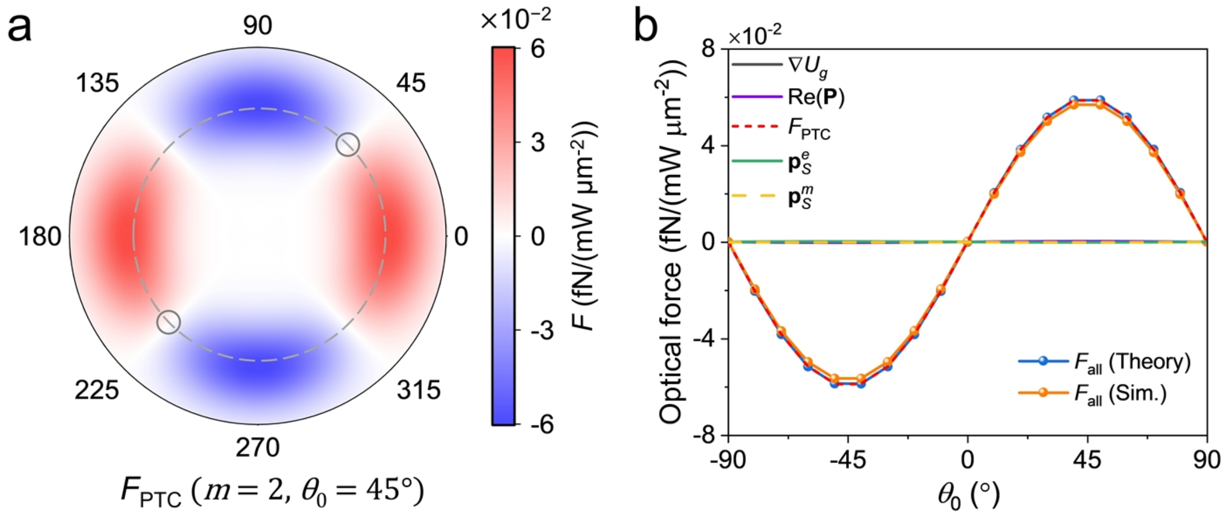

Figure S8. Simulation of optical forces of the vector beam after focusing. (a) Simulated F_{PTC} on the 100-nm polystyrene nanoparticle using the Minkowski stress tensor in the focused beam when m = 2 and θ_0 = 45°. (b) Different types of optical forces on the 100-nm nanoparticle under various polarization angles when m = 2. Forces from the intensity gradient (∇U_g), the real part of the Poynting momentum [Re(**P**)] and the Belinfante spin momentum ($\mathbf{p}_S^e$ and $\mathbf{p}_S^m$) vanish due to the $|E|$ symmetry. The PTOF increases after focusing, which can be attributed to reduction of the radius of the ring and the corresponding enhancement of the polarization gradient.

The focused beam retains the polarization of the incident beam while acquiring a radial phase gradient, as shown in Fig. S7. Consequently, PTOF emerges as the dominant force along the angular direction of the annular beam, as illustrated in Fig. S8, wherein optical forces arising from spin momentum and spin-orbit interaction vanish due to the symmetry of $|E|$. This observation corroborates the established understanding that spin momentum and spin-orbit interaction rely on inhomogeneous spin angular momentum (SAM), such as circular-polarization optical beams, interfaces, asymmetric scattering, or oblique incidence. In experiments, the optical gradient force manifests as pushing particles toward regions of higher field intensity. Other optical forces lack a direct experimental criterion and thus require simulation and comparative analysis based on measured field parameters to ascertain their respective contributions within the specific experimental context, as demonstrated in Figs. S6a and S8b.

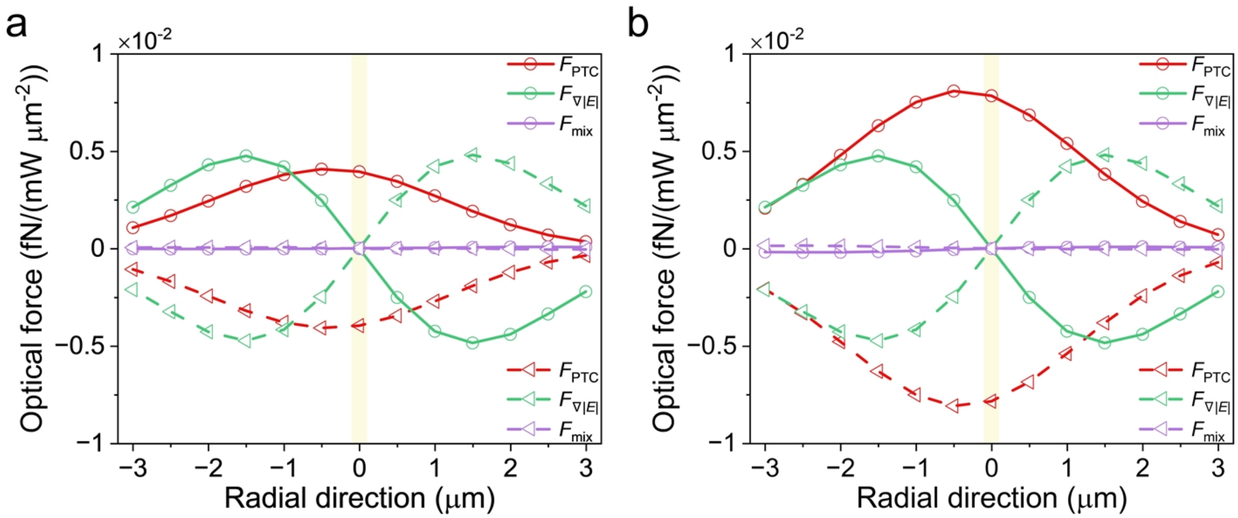

Figure S9. Comparison of the PTOF, $\boldsymbol{F}_{\nabla|\boldsymbol{E}|}$ and $\boldsymbol{F}_{\mathbf{mix}}$. Simulation of the PTOF, $F_{\nabla|E|}$ and F_{mix} when (a) m = 1 and (b) m = 2. At the "0" point (maximum-intensity point), $F_{\nabla|E|}$ and F_{mix} vanish, meaning that only the PTOF exhibits in the circle. $F_{\nabla|E|}$ is independent of the polarization gradient, thus independent of the topological charge, resulting in the same magnitude for m = 1 and m = 2. Polarization angles θ are 45° and −45° for solid and dashed curves, respectively.

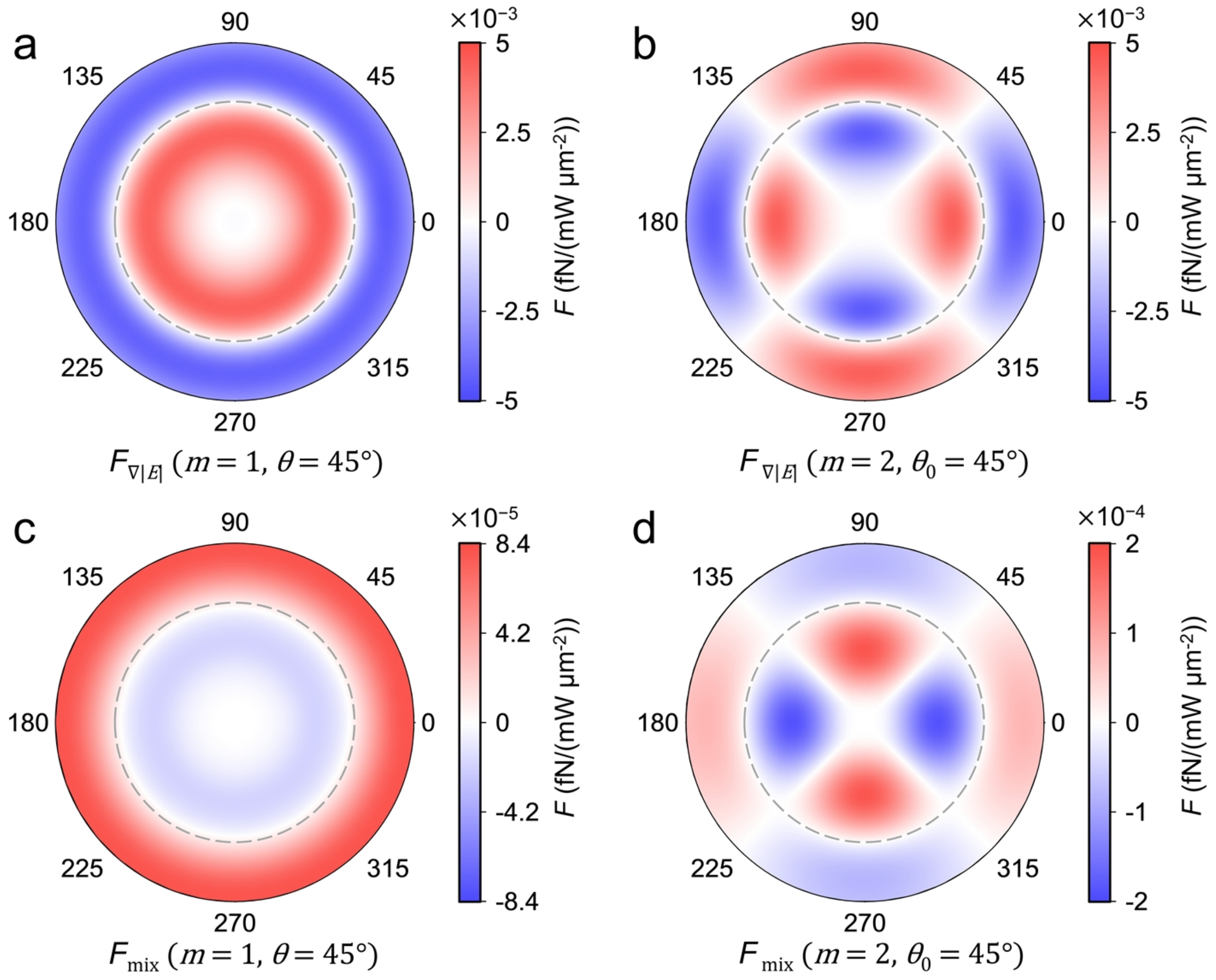

Figure S10. Simulation of $F_{\nabla|E|}$ and F_{mix} from the imaginary Poynting momentum. Simulated $F_{\nabla|E|}$ on the 100-nm nanoparticle using the Minkowski stress tensor in the doughnut-shaped beam when (a) $m = 1$, $\theta = 45°$ and (b) $m = 2$, $\theta_0 = -45°$. Simulated F_{mix} on the 100-nm nanoparticle when (c) $m = 1$, $\theta = 45°$ and (d) $m = 2$, $\theta_0 = -45°$. $F_{\nabla|E|}$ and F_{mix} are in opposite directions on both sides of the light beam due to the opposite intensity gradient, and vanish at the maximum-intensity point. $F_{\nabla|E|}$ and F_{mix} are both dependent on the polarization, therefore exhibit topological properties.

The PTOF, $F_{\nabla|E|}$, and F_{mix} are respectively associated with $\mathrm{Im}(\mathbf{\Pi})_{PTC}$, $\mathrm{Im}(\mathbf{\Pi})_{\nabla|E|}$, and $\mathrm{Im}(\mathbf{\Pi})_{mix}$. It is noteworthy that all three types of forces depend on both the polarization state and the amplitude, thus exhibit topological properties. $F_{\nabla|E|}$ is independent of the polarization gradient, yielding identical magnitudes for $m = 1$ and $m = 2$ under the same polarization angle. At the point of maximum intensity, only the PTOF manifests, aligning well with experimental observations.

Owing to the opposite intensity gradient, $F_{\nabla|E|}$ and F_{mix} are oppositely directed on either side of the beam and vanish at the intensity maximum. In contrast, the PTOF maintains the same direction on both sides.

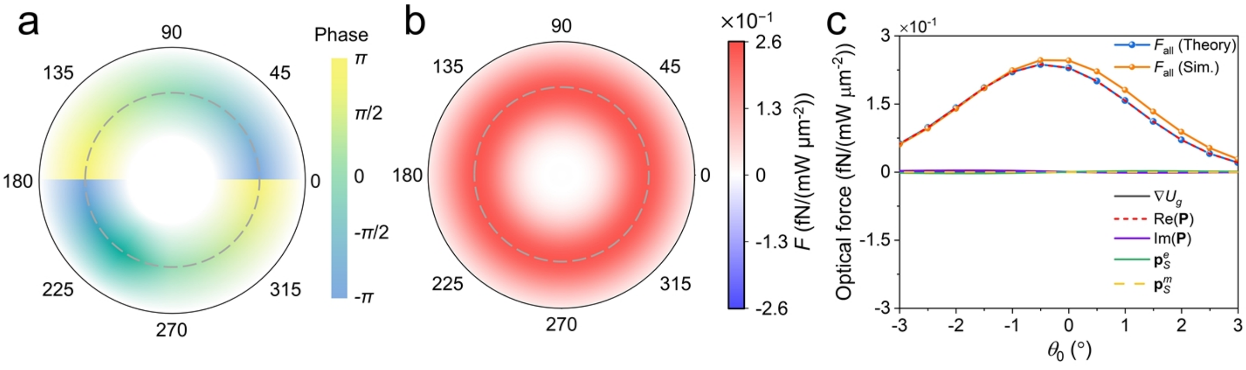

Figure S11. Simulation of optical forces of the vortex beam. Simulated total optical force (b) and different types of optical forces (c) on the 100-nm polystyrene nanoparticle under the left-circularly polarized vortex beam with topological charge $n = 2$ (a) [simulation radius, 9 μm; radius of the circle with the maximum intensity (dashed line), 6 μm]. Forces from the intensity gradient (∇U_g) vanish due to the $|E|$ symmetry in the azimuthal direction. The dominant force originates from the real part of the Poynting momentum [Re(**P**)], which manifests here as the phase gradient force. Due to the presence of an intensity gradient in the radial direction, forces associated with the IPM and the Belinfante spin momentum ($\mathbf{p}_S^e$ and $\mathbf{p}_S^m$) are also present, though they are approximately two orders of magnitude weaker than the phase gradient force.

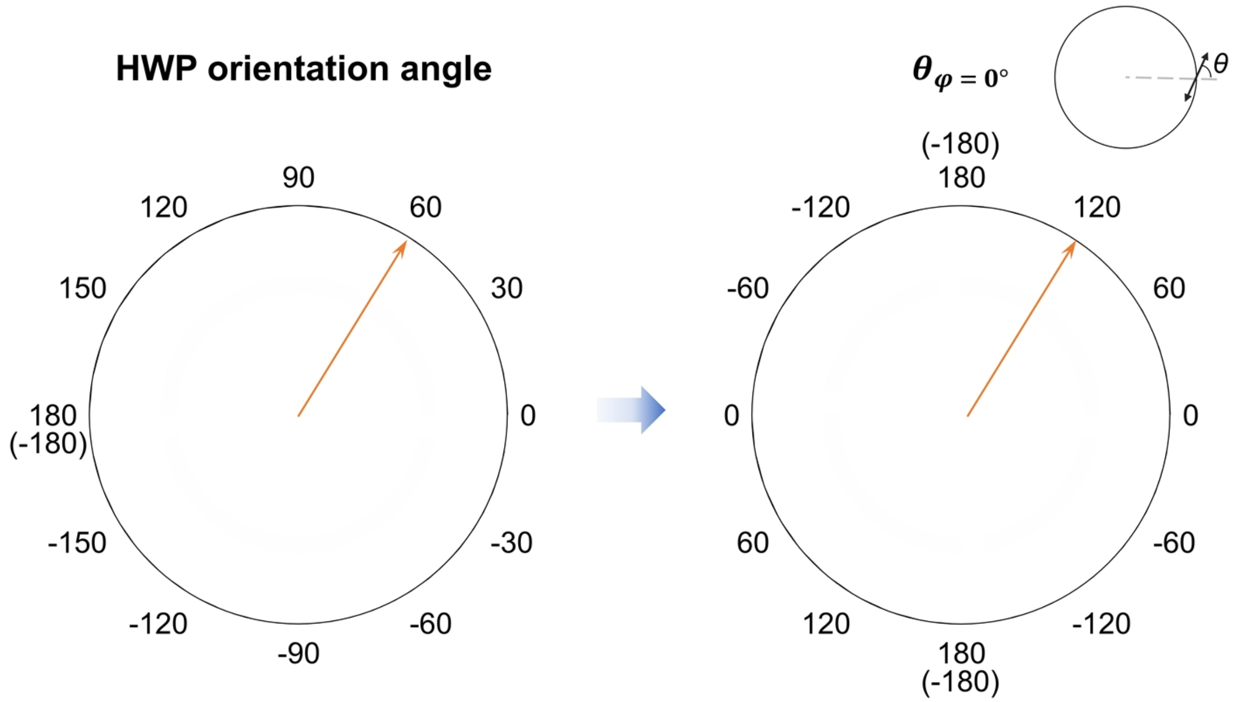

Figure S12. Control of the polarization angle in the vector beam using the half-wave plate. The orientation of the half-wave plate (HWP) can change the polarization angle θ accordingly. The polarization angle has a linear dependence on the rotation angle of the HWP. For instance, a 60° rotation of the HWP can produce a polarization angle of 120° at $\varphi = 0°$. The laser intensity in the circle is set to 1 mW/μm^2.

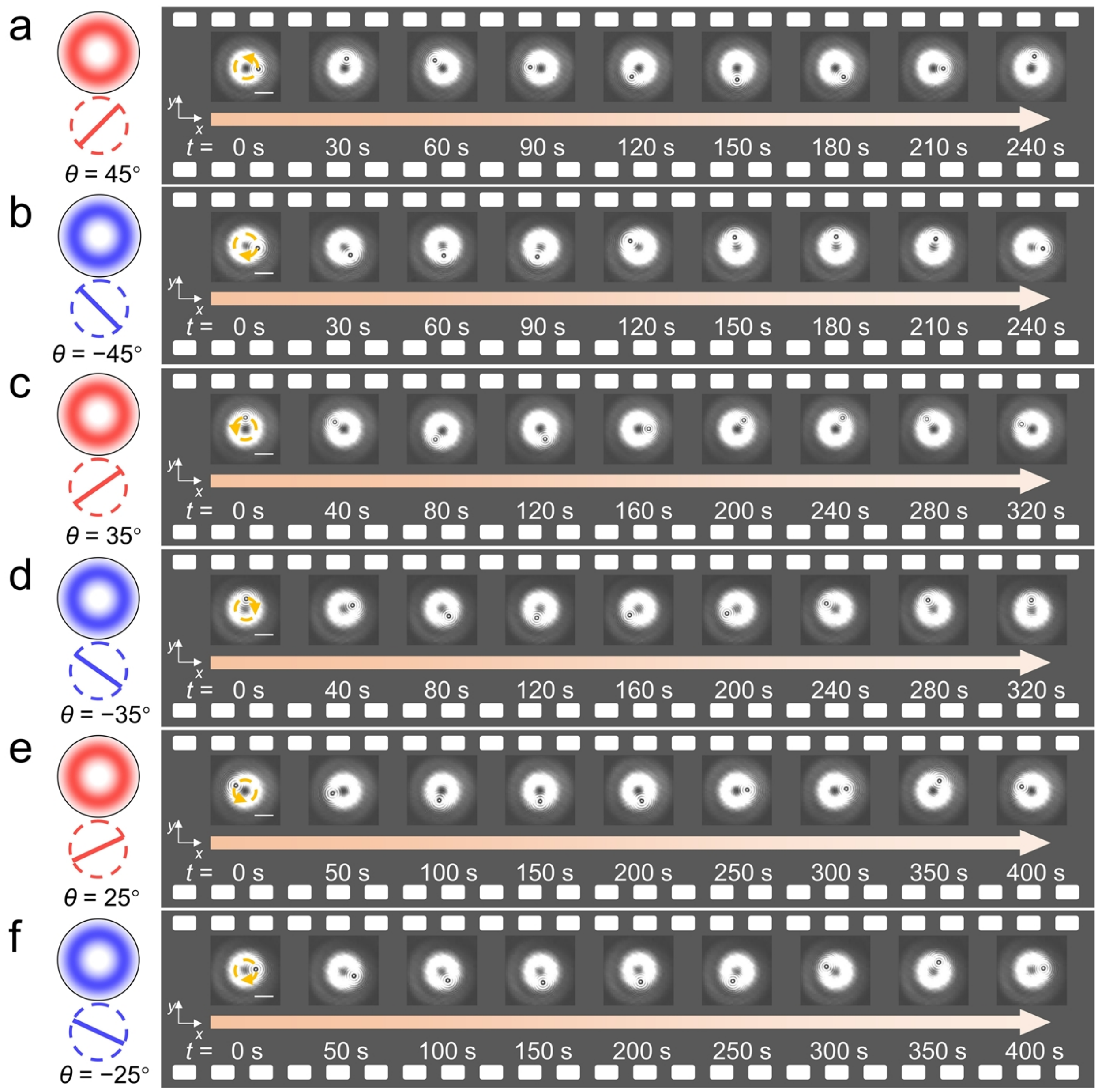

Figure S13. Experimental observation of the particle rotation by the imaginary Poynting momentum when *m* = 1. 3-μm particles rotate counter-clockwise and clockwise when $\theta > 0°$ and $\theta < 0°$, respectively. Particle velocity and optical forces vary with different polarization angles θ (see Fig. 5a). Scale bars equal 10 μm. The laser intensity in the circle is measured to be 0.79 mW/μm^2.

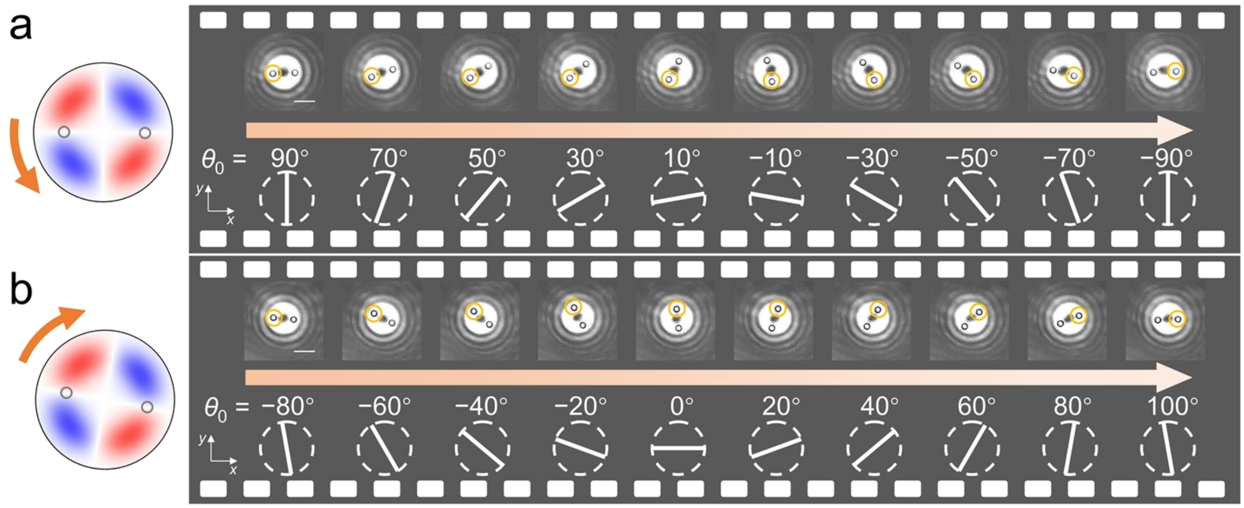

Figure S14. Dynamics of the particle pair by the imaginary Poynting momentum when m = 2. A particle pair emerges when $m = 2$. The particle pair can rotate clockwise or counter-clockwise depending on different orientation angles of the half-wave plate. Scale bars equal 10 μm.

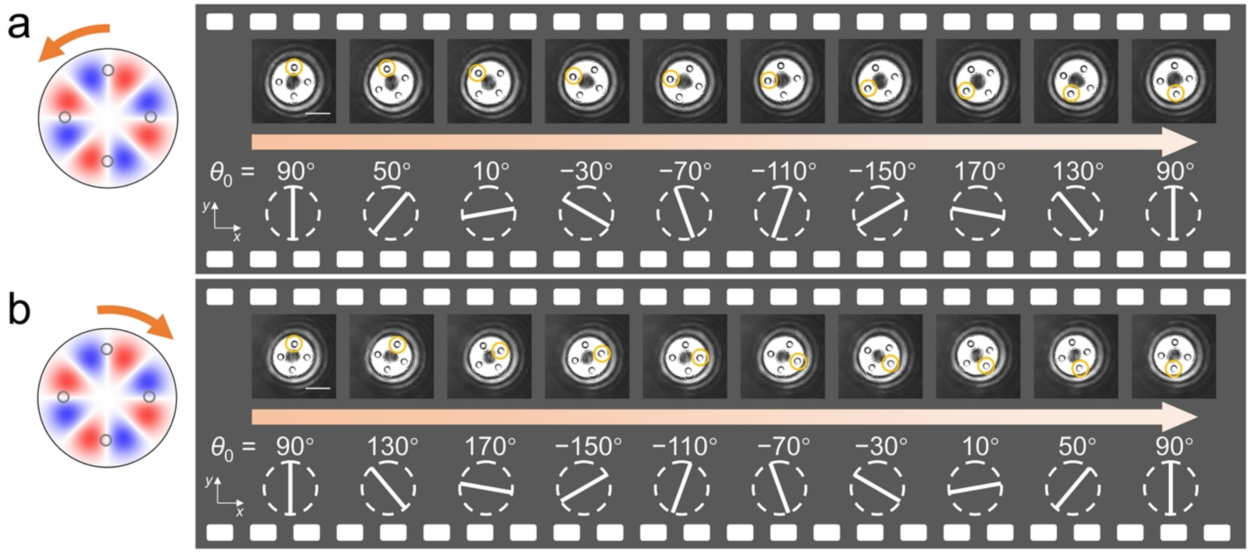

Figure S15. Dynamics of the four-particle array by the imaginary Poynting momentum when *m* = 3. Four particles are uniformly distributed along the circle and can be rotated by adjusting the orientation of the half-wave plate. Scale bars equal 10 μm.

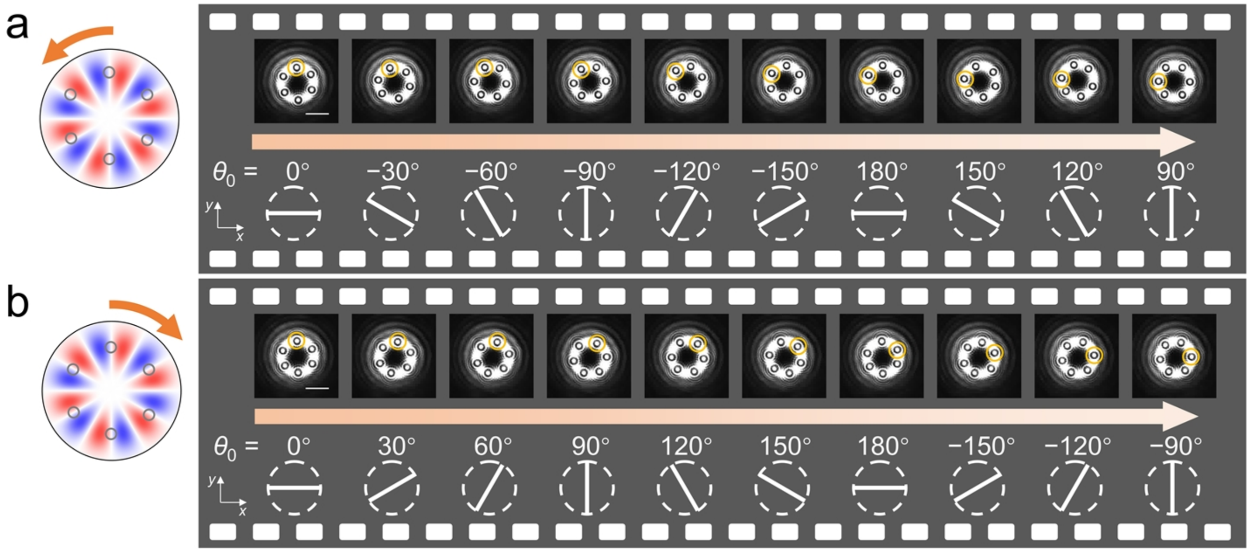

Figure S16. Dynamics of the six-particle array by the imaginary Poynting momentum when *m* = 4. Six particles are uniformly distributed along the circle and can be rotated by adjusting the orientation of the half-wave plate. Scale bars equal 10 μm.

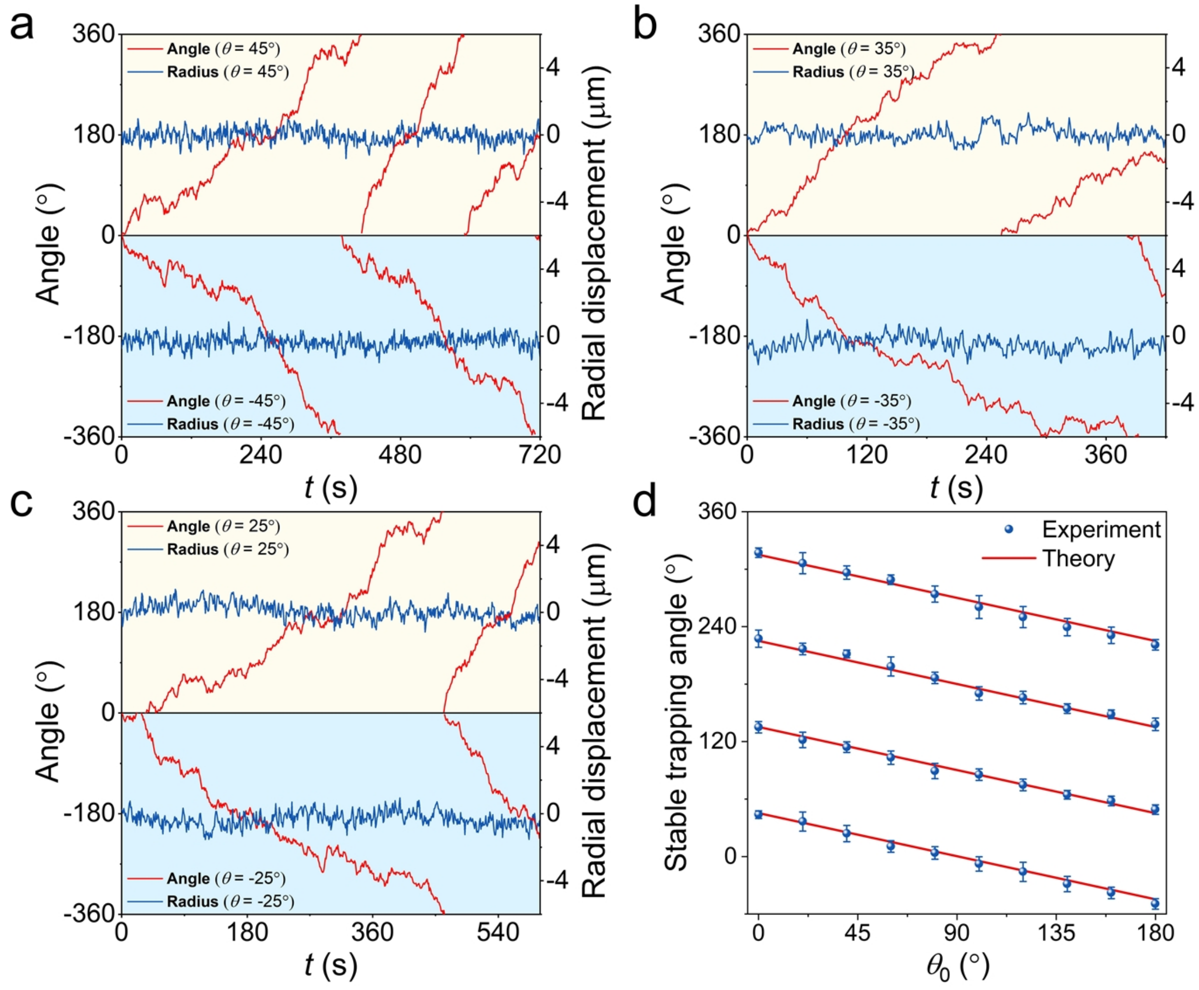

Figure S17. Particle rotation angles by the imaginary Poynting momentum. (a−c) Rotation angles of particles for different θ when $m = 1$. Particles are confined to intensity maximum positions ("$x = 0$" positions, also the "circle" in the main text) and rotate smoothly with time. (d) Four stable trapping angles emerge at a given θ (at $\varphi = 0°$) when $m = 3$. The particle array rotates continuously with the polarization angle, showing the convincing evidence of the high-order topology of the IPM.
